# A balance of metabolism and diffusion articulates a gibberellin hormone gradient in the *Arabidopsis* root

**DOI:** 10.1073/pnas.2425320122

**Published:** 2025-11-24

**Authors:** Kristian B. Kiradjiev, Jayne Griffiths, Alexander M. Jones, Leah R. Band

**Affiliations:** ^a^Centre for Mathematical Medicine and Biology, School of Mathematical Sciences, University of Nottingham, Nottingham NG7 2RD, United Kingdom; ^b^Sainsbury Laboratory, University of Cambridge, Cambridge CB2 1LR, United Kingdom; ^c^Division of Plant and Crop Sciences, School of Biosciences, University of Nottingham, Sutton Bonington LE12 5RD, United Kingdom

**Keywords:** gibberellin, multiscale modeling, plant hormones, *Arabidopsis* root development, hormone biosensor

## Abstract

The plant hormone gibberellin (GA_4_) regulates numerous developmental processes. Within the root, GA_4_ controls growth, in part, by controlling the extent of cell elongation. The nlsGPS1 FRET biosensor revealed a GA_4_ gradient within the *Arabidopsis* root growth zones, with GA_4_ levels correlating with cell length. We developed a multiscale mathematical model to understand how biosynthesis, catabolism, and transport create the GA_4_ distribution within the root growth zones. The model predicted that phloem delivery of the biosynthetic intermediate GA_12_ contributes to higher levels of bioactive GA_4_ in the elongation zone, with the GA_4_ synthesis pattern being further modified by local GA_12_ synthesis in the quiescent center region and the spatial distribution of biosynthesis enzymes (GA20ox and GA3ox). Model predictions suggested that while GA20ox and GA3ox transcript is present throughout the growth zones, these enzymes are inactive in the dividing cells, which explains steep GA_4_ gradients observed in the GA3ox overexpression line and improves agreement between model predictions and data in wildtype. The model suggested that the GA_4_ gradient also depends on a balance of diffusion through plasmodesmata and catabolism. Both model predictions and biosensor data demonstrated that plasmodesmatal diffusion enables a more gradual GA_4_ gradient, with higher diffusion antagonizing the GA_4_ gradient. Model predictions suggested that catabolism limits GA_4_ levels, which we validated via biosensor imaging in the *ga2oxhept* mutant. In conclusion, our results suggest that local GA_4_ synthesis combines with diffusion and catabolism to create a spatial GA_4_ gradient that provides positional information and patterns cell elongation.

## Significance

Many aspects of animal and plant development are controlled by the spatial distribution of small molecules, termed hormones. In plants, the hormone gibberellin (GA_4_) controls key developmental processes including root growth. By developing a mathematical model, we investigated how the GA_4_ distribution in the root arises. Comparing the model predictions with experimental biosensor data suggested that the GA_4_ distribution depends on distribution of enzyme activities combined with GA_4_ diffusion between adjacent cells through small channels called plasmodesmata. The knowledge and tools gained here will be valuable for understanding how GA_4_ controls growth as well as investigating the spatial distributions of other hormones that influence plant development.

The plant hormone gibberellin (GA_4_) controls numerous developmental processes and environmental responses. In *Arabidopsis thaliana*, GA_4_ controls root growth by regulating both cell production in the meristem (the region of dividing cells close to the root apex) (1, 2), and cell growth in the adjacent elongation zone (EZ) (where cells undergo rapid elongation) (3). It is well established that plants with reduced GA_4_ biosynthesis (due to mutations in key enzymes) have reduced root growth, due to both a shorter meristem (1, 2) and a shorter elongation zone (3); however, we lack mechanistic understanding of how these mutations affect the GA_4_ distribution and how the GA_4_ distribution mediates the growth responses.

The GA_4_ FRET biosensor, nlsGPS1, revealed a GA_4_ gradient within the *Arabidopsis* root growth zone, with GA_4_ levels being lowest in the division zone and increasing as cells begin rapid elongation (4). To understand the mechanisms controlling this root GA_4_ gradient, we created an initial multicellular mathematical model of GA_4_ dynamics within the root growth zones (5). The model predicted that the GA_4_ gradient is due to low biosynthesis in the meristem, and an increase in biosynthesis toward the shootward end of the meristem (5). While this initial model provided insights into a necessary differential in biosynthesis, it remained unclear how this relates to the distribution of GA_4_ metabolism enzymes and whether cell-to-cell transport also influences the GA_4_ gradient.

There are numerous cell-scale processes that may affect the GA_4_ distribution across the root growth zone. Bioactive GA_4_ is synthesized through a series of oxidation steps, mediated by GA20oxidases (GA20ox) and GA3oxidases (GA3ox), from the precursor GA_12_. This GA_12_ is both delivered to the root via the phloem (6) and locally synthesized (5). The enzymes involved in the downstream GA_4_ metabolism (i.e. the combination of biosynthesis and catabolism) exhibit spatial variations in expression level (5, 7) which may lead to GA_4_ gradients. In addition, hormone distributions are influenced by transmembrane transport. Recent studies revealed that gibberellin metabolites are actively transported across cell membranes via NPF and SWEET proteins (8–10). Furthermore, hormones diffuse between adjacent cells through plasmodesmata, which has been shown to influence hormone-related phenotypes (11), though how plasmodesmatal diffusion contributes to GA_4_ distribution is currently unknown.

While the nlsGPS1 FRET biosensor has shown how perturbing the GA_4_ biosynthesis enzymes affects the GA_4_ gradient in the root growth zones (5), modeling is essential to infer how the GA_4_ gradient arises from dynamic processes at the cellular scale. By developing a multiscale mathematical model, we now investigate how metabolism and transport combine to articulate the GA_4_ gradient. The model predicts how the GA_4_ gradient emerges from the combination of i) GA_12_ delivered to the EZ via the phloem together with locally synthesized GA_12_, ii) spatial patterns of GA20ox and GA3ox biosynthesis enzyme activity, iii) plasmodesmatal diffusion, and iv) catabolism. The model predictions are refined and validated using FRET biosensor data in wildtype and lines with perturbed metabolism enzymes. Thus, our study suggests that GA_4_ distributes analogously to a classic “morphogen” reminiscent of Wolpert’s French Flag pattern (12), with localized synthesis combining with diffusion and catabolism to create a spatial gradient that underlies growth regulation.

## Results

### Model Description.

**Fig. 1.**
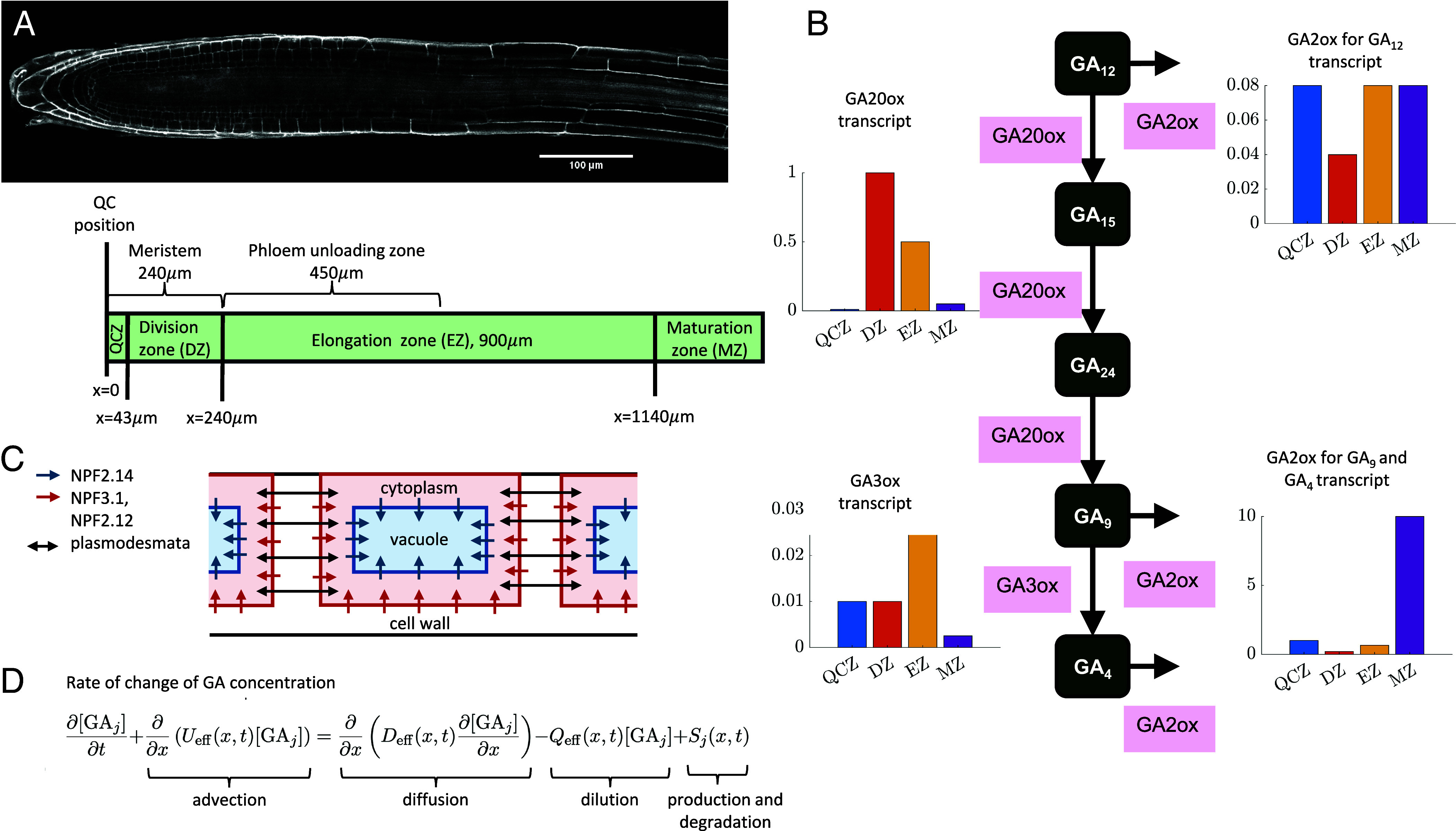
(*A*) Confocal image of the *Arabidopsis* root growth zones, together with a to-scale schematic of the modeled domain, showing the positions of the different zones. The model is one-dimensional, with x=0 positioned at the quiescent center (QC). (*B*) Schematic of the pathway downstream of GA_12_ that mediates the biosynthesis and catabolism of GA_4_, together with data showing the relative transcript levels of the enzymes, GA20ox, GA3ox, and GA2ox (showing separately the levels for GA2ox mediating GA_12_ degradation and for GA2ox mediating GA_9_ and GA_4_ degradation) (5, 7). (*C*) Schematic focusing on a single cell showing the cell compartments, the transport components, and the localization of the NPF transporters. (*D*) Continuum governing equations for each metabolite, j=12,15,24,9, and 4. The equations for each metabolite are coupled through their production and degradation terms, $S_{j}\left(x,t\right)$, which represent the metabolic pathway depicted in Fig. 1*B*, and correspond to formulae given in *SI Appendix*, section 5.

To investigate the GA_4_ distribution in the *Arabidopsis* root growth zone, we created a cell-based mathematical model that predicts the GA_4_ concentration in a cell file. One end of the file is situated at the quiescent center (QC), and the other is within the maturation zone. Thus, the file comprises the meristem, where cells elongate slowly and divide, the elongation zone (EZ), where cells rapidly elongate, and a portion of the maturation zone (MZ), where cells have ceased elongation (Fig. 1*A*). We modeled mature roots in which the sizes of the meristem and EZ are stable. In the simulations, the cell elongation and division rates are prescribed: Cell elongation is prescribed from previous experimental data on the relative elongation rates (RER) (13) (*SI Appendix*, Fig. S1 and Table S1); in the meristem, we assumed that cells divide once they have approximately doubled in size, prescribing initial cell lengths based on measurements in ref. 14. When a cell reaches the end of the meristem (due to growth of the more rootward cells), it ceases division and begins rapid elongation.

We simulated the GA_4_ concentration in each cell cytoplasm, cell vacuole, and intercellular apoplastic compartment (*SI Appendix*, Fig. S2) [noting that the vacuole has recently been suggested to contribute to GA_4_ distribution (10)]. Within the meristem, the cells’ vacuolar volume fraction is constant [approximately 35% (15)], requiring both the cytoplasm and vacuole to expand with a relative growth rate equal to the RER of the cell. In the EZ, it is predominantly the vacuole that expands (15, 16). Previous root models assumed that the cell elongation in the EZ was entirely due to vacuolar expansion (5, 17); however, recent data suggest some cytoplasmic expansion in the EZ (15, 16). Our calculations revealed that these data (15, 16) are consistent with the cytoplasm continuing to expand at the slower “meristem” rate throughout the EZ, with a vacuolar expansion rate approximately five times higher (*SI Appendix*, section 8); such expansion results in a vacuolar volume fraction of 90% once cells enter the maturation zone.

We modeled GA_4_ metabolism within every cell. GA_4_ biosynthesis involves a series of oxidation steps, converting the precursor, geranylgeranyldiphosphate, to bioactive GA_4_ (18, 19). GA_4_ biosynthesis has been shown to be predominantly regulated at the later steps of this pathway, whereby GA_12_ is converted to bioactive GA_4_ (20). Thus, in every cell, we modeled the network (Fig. 1*B*) whereby GA_12_ undergoes a series of oxidation steps mediated by GA 20-oxidase (GA20ox) to produce GA_9_, which is converted to the bioactive GA_4_ by GA 3-oxidase (GA3ox) (18). The steps in the GA_4_ biosynthesis pathway are modeled via Michaelis–Menten kinetics for the metabolic reactions (21); parameter values for GA20ox-mediated steps are estimated in ref. 21 using data from ref. 22, and parameter values for the GA3ox-mediated step are estimated in ref. 23. GA 2-oxidase (GA2ox) enzymes mediate catabolism, which we model via linear terms (24). Here, we consider two groupings of the GA2oxidase family: GA2oxidases which mediate catabolism of GA_9_ and GA_4_ (AtGA2ox1, AtGA2ox2, AtGA2ox3, AtGA2ox4, AtGA2ox6), and GA2oxidases which mediate the catabolism of GA_12_ (AtGA2ox7, AtGA2ox8, AtGA2ox9, AtGA2ox10) (25). Transcriptomics data (5, 7) suggest levels of GA20ox, GA3ox, and GA2ox vary across the root growth zones (Fig. 1*B*), and similar trends are seen in ref. 26. Assuming the enzyme activities correlate with the transcript levels, we prescribed enzyme rates depending the cell’s zone, further subdividing the meristem into the quiescent-center zone (QCZ, 0 to 43 μm from the QC) and division zone (DZ, between 43 to 240 μm from the QC), based on the data in ref. 5, 7 (Fig. 1*A*). While expression of most early GA_4_ biosynthetic enzymes extends throughout the root growth zone, the first committed step in GA_12_ synthesis, ent-copalyl diphosphate synthase (CPS), exhibits high expression close to the QC and low expression in the DZ and EZ (5), and thus we assumed that GA_12_ is locally synthesized in the QCZ. Furthermore, we assumed that GA_12_ is delivered in the phloem-unloading zone [the rootward half of the EZ (27)], assuming, for simplicity, that GA_12_ delivery is spatially uniform within this zone. Inclusion of GA_12_ delivery is motivated by grafting studies which suggested that GA_12_ is the main mobile gibberellin and is transported from shoot-to-root within the phloem (6). These studies used the *kao1kao2* line which lacks the final step in GA_12_ synthesis and showed that grafting a wildtype shoot to a *kao1kao2* mutant root partially restored root growth compared with using a *kao1kao2* shoot (that lacks GA_12_ synthesis everywhere) (6). These data provide evidence that root growth utilizes both locally synthesized GA_12_ and GA_12_ delivered from the shoot: Our model enables us to assess the relative roles of these two GA_12_ sources.

To investigate the role of transport, for each gibberellin metabolite in the modeled pathway, we incorporated passive diffusion across the plasma membrane and tonoplast, and active transport via membrane carriers of the NPF family, considering influx carriers (NPF3.1 and NPF2.12), to be on the plasma membrane (importing from the apoplast to the cytoplasm) and an efflux carrier, NPF2.14, on the tonoplast (exporting from the cytoplasm to the vacuole) (9, 10) (Fig. 1*C*). We note that, in the root growth zone, these NPF carriers are localized on specific cell types [NPF3.1 in the endodermal plasma membranes (9), and NPF2.12 and NPF2.14 in the pericycle plasma membranes and tonoplasts, respectively (10)], and we therefore consider simulations with and without these transporters in our model to assess whether the presence of these transporters affects the predicted gradients. We note that SWEET transporters (8) are not modeled, as within the root, transcriptomics data suggest expression only in the stele in the late EZ and maturation zone (28). We further made the simplifying assumption that each gibberellin metabolite concentration is modeled as spatially uniform within each cell cytoplasm and vacuole; noting that intracellular diffusion is sufficient to smooth out intracellular gradients within the smaller cells in the meristem, but intracellular gradients may be present within the elongated cells further from the root tip (28), see *SI Appendix*, Table S4.

As for other plant hormones, only protonated metabolites can passively diffuse into cells, whereas the NPF carriers transport the anionic forms (10, 29). In the acidic apoplast, ∼7% of GA_4_ is protonated and able to passively diffuse into cells, with the remaining 93% being anionic requiring influx via NPF3.1 and NPF2.12 (30). In the cytoplasm over 99% of GA_4_ is anionic, and so efflux is predominantly via the NPF2.14 carriers for cytoplasmic export into the vacuole (30). Transporter permeabilities are estimated from oocyte data (9, 10, 31). These estimates suggested that protonated GA_12_, GA_15_, and GA_9_ diffuses passively across cell membranes faster than protonated GA_24_ and GA_4_ but the corresponding anionic forms have slower NPF-mediated active transport (*SI Appendix*, Table S2); these differences are thought to be due to the specific structure of the metabolites (31).

The model included diffusion within the apoplast (32), which provides an alternative continuous pathway for hormone diffusion (29). The model incorporated diffusion between adjacent cells through plasmodesmata (11, 33), which our previous generic-cell-file model suggested may be a significant pathway for GA_4_ movement (30).

The above model assumptions are represented by a system of ordinary differential equations (ODEs) for the concentration of each gibberellin metabolite in every cellular compartment, as detailed in *SI Appendix*, sections 1–5.

### Continuum Approximation Can Reproduce the Cell-Based Model Solutions.

To gain insight into the underlying mechanisms and reduce the model’s computational cost, we derived a continuum approximation of the above cell-based model. The approach enables us to relate the discrete cell-based model to an equivalent macroscale continuum model, where, instead of tracking concentrations in individual cellular compartments, we introduce macroscale concentration variables continuously varying with distance.

We previously derived a continuum approximation of a simple GA_4_ model (30), which considered transport in a single file of growing identical cells (with no synthesis and degradation). This analysis showed that GA_4_ transport in a cell file can be represented by a continuum model, whereby GA_4_ concentration is a function of time and distance and the GA_4_ dynamics are governed by a single reaction–advection–diffusion equation.

Building on this previous analysis (30), we derived a continuum approximation of GA_4_ transport and metabolism across the three root growth zones (meristem, EZ, and maturation zone) (*SI Appendix*, sections 1–4 and Fig. S3). In each zone, the analysis led to a system of coupled partial differential equations, i.e. one equation for each metabolite, that contains terms representing advection, diffusion, dilution, and production/degradation (Fig. 1*D*), providing formulae for the effective velocity, diffusivity, and dilution rate, in terms of the parameters governing the cell-scale metabolism, transport, and cell-growth dynamics. These formula revealed that plasmodesmatal diffusion, apoplastic diffusion, and NPF-mediated transport contribute to the diffusion terms, whereas cell growth and spatial variance in the cell lengths and the subcellular compartmentalization lead to the advection and dilution terms. The systems of equations representing the dynamics in each growth zone are then coupled at the boundaries between the three zones. We note that a continuum approximation of GA_1_ dynamics in the Maize leaf growth zone was previously presented in ref. 34, albeit with no transport between adjacent cells which resulted in a much simpler derivation and no diffusion terms.

The continuum approximation greatly simplified the computational process of simulating the model, enabling us to introduce more complex dynamics and perform more detailed parameter surveys. The continuum approximation also provided insights into the model dynamics via the analytic expressions for key effective parameters, which revealed how the cell-scale processes contribute to the effective diffusivity, induced hormone velocity, and dilution rate.

### Model Predictions Can Reproduce GA_4_ Gradient Observed Using the nlsGPS1 Sensor.

The model predicts the distribution of the modeled gibberellin metabolites along the root growth zones (considering the steady-state distributions). Having predicted the GA_4_ distribution, the corresponding distribution of the nlsGPS1 emission ratio is calculated using the nonlinear relationship suggested from titration curves in ref. 4. GIBBERELLIN PERCEPTION SENSORS are fluorescent biosensors that directly interact with bioactive GA_4_ via a sensory domain derived from the *Arabidopsis* GA_4_ perception machinery (4, 35). The fluorescence emission ratios of the biosensors quantitatively report on GA_4_ concentrations in solution, for nlsGPS in nuclei.

Estimates for most model parameters are available in literature (*SI Appendix*, Tables S1–S3). However, the synthesis/delivery rates for GA_12_ and degradation rates for GA_12_, GA_9_, and GA_4_ are unknown. To identify appropriate values for these parameters, we performed a parameter survey comparing the predicted distribution of the nlsGPS1 emission ratio with experimental biosensor data (*SI Appendix*, section 6 and Fig. S4). The experimental biosensor datasets analyzed here were primarily emission ratios from the outer cell layers of the root growth zones (using data previously published by Rizza et al. (5), from nuclei 0 to 500 microns from the root tip) and the model considers these cell layers for parameterization. The parameter survey suggests that the degradation rates for GA_12_, GA_9_, and GA_4_ are approximately equal, and that the rate of GA_12_ delivery via the phloem is substantially larger than the rate of GA_12_ synthesis in the QCZ (*SI Appendix*, Table S5).

**Fig. 2.**
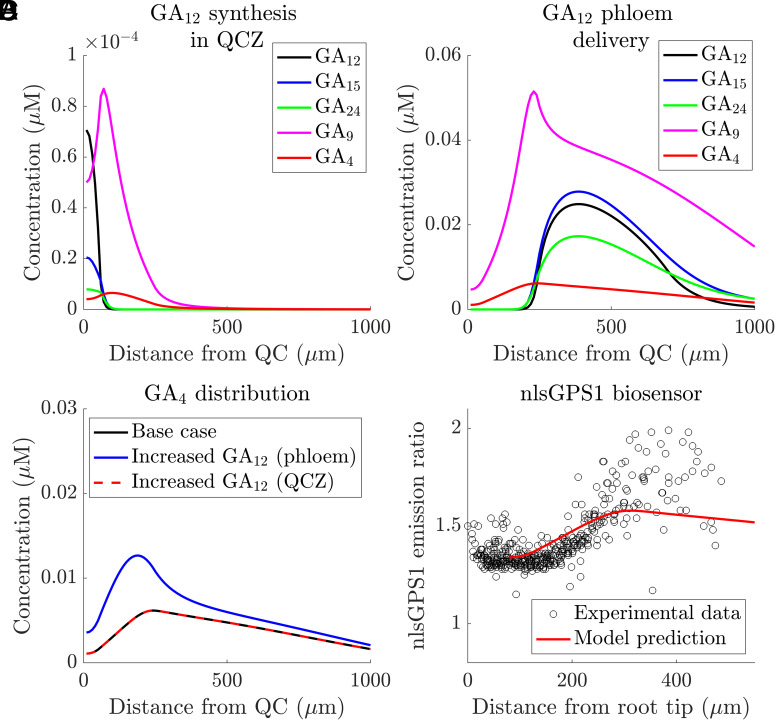
Model predictions can reproduce GA_4_ gradient observed using the nlsGPS1 sensor. (*A* and *B*) Predicted GA_12_, GA_15_, GA_24_, GA_9_, and GA_4_ distributions due to (*A*) local GA_12_ synthesis in the QCZ, and (*B*) GA_12_ delivery in the phloem-unloading zone. (*C*) Predicted GA_4_ distribution (due to both GA_12_ delivery in the phloem-unloading zone), together with predictions in which either the GA_12_ synthesis rate in the QCZ is doubled, or the GA_12_ delivery rate in the phloem-unloading zone is doubled. (*D*) Comparison between the predicted and observed distribution of nlsGPS1 emission ratios. Predicted sensor distribution is calculated from the base case GA_4_ distribution shown in panel (*C*). The experimental data are reproduced from figure 1F in ref. 5 giving nlsGPS1 emission ratios from nuclei between 0 to 500 μm from the root tip. Quantification of the difference between the predictions and data, Mean-Squared-Error, MSE=0.0154 (*SI Appendix*, section 7).

The GA_12_ locally synthesized in the QCZ is predicted to create a GA_4_ distribution with a peak within the DZ (Fig. 2*A*). The GA_12_ delivered in the phloem-unloading zone is predicted to generate substantially higher GA_4_ levels, with GA_4_ levels increasing as cells transition the meristem (Fig. 2*B*). Combining both sources of GA_12_ results in a superposition of the two GA_4_ distributions, with the phloem-delivered GA_12_ making a larger contribution (Fig. 2*C*). The corresponding predicted nlsGPS1 distribution agrees well with the experimental data (Fig. 2*D*). As expected, the model predicts that increasing either the rate of GA_12_ synthesis in the QCZ or the rate of GA_12_ delivery via the phloem increases the GA_4_ levels (*SI Appendix*, Fig. S5); albeit, with increasing the GA_12_ delivery rate having a more substantial effect on the overall GA_4_ levels (Fig. 2*C*).

We used the model to evaluate the roles of the spatial distributions of both GA_12_ synthesis/delivery and enzyme levels. The model predicted that the GA_4_ distribution relies on the distribution of GA_12_ synthesis/delivery. With a uniform GA_12_ synthesis/delivery, the model does predict a GA_4_ gradient (due to the enzyme transcript distribution); however, this gradient is closer to the root tip with a wide peak within the DZ, inconsistent with the sensor data (*SI Appendix*, Fig. S6). In addition, the enzyme distributions are also essential for the predicted GA_4_ gradient—with uniform enzyme levels, the predicted distributions of all the gibberellin metabolites peak where GA_12_ is delivered/synthesized, resulting in model predictions which overestimate the GA_4_ levels within the EZ (*SI Appendix*, Fig. S7).

In conclusion, the model predictions can capture the GA_4_ gradient observed using the nlsGPS1 biosensor, and suggest that the GA_4_ gradient is due to the combined effect of GA_12_ delivery and spatial variations in the metabolism enzymes. The model suggests that GA_12_ delivered to the root through the phloem is an important contributor to the GA_4_ gradient.

### Enzyme Inactivation in the Division Zone Is Essential to Explain the GA_4_ Distributions with GA20ox and GA3ox Overexpression.

**Fig. 3.**
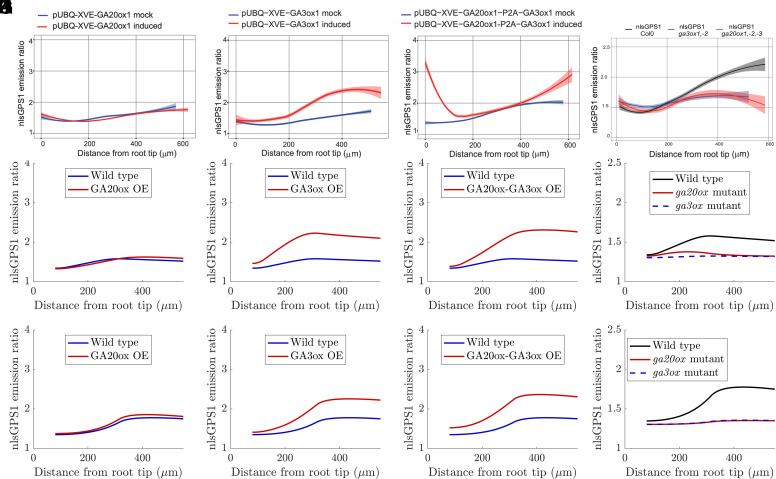
Enzyme inactivation in the division zone is essential to explain the sensor distributions in the GA20ox and GA3ox overexpression. (*A*–*D*) Experimental nlsGPS1 distributions reproduced from figure 1B and figure 2B,D,E in ref. 5. (*E*–*L*) Predicted nlsGPS1 emission ratio of GA_4_ for (*E* and *I*) GA20ox overexpression, (*F* and *J*) GA3ox overexpression, (*G* and *K*) both GA20ox and GA3ox overexpression, and (*H* and *L*) *ga20ox* and *ga3ox* mutants. (*E*–*H*) Model predictions with enzyme activities based on transcript levels (shown in Fig. 1*B*). (*I*–*L*) Model predictions in which the GA20ox and GA3ox activity is set to zero in the DZ (with all other enzyme activities based on the transcript level). Model predictions with alternative assumptions are provided in *SI Appendix*, Fig. S8. Quantification of the difference between the predictions and data are provided in *SI Appendix*, Table S6.

Overexpressing GA3ox enzyme was observed to significantly increase the nlsGPS1-detected GA_4_ in the EZ and the slope of the gradient, whereas overexpressing GA20ox enzyme had little effect (5) (Fig. 3 *A* and *B*). Consistent with these observations, the model predicted that overexpression of GA3ox resulted in a larger change in nlsGPS1 emission ratios compared with overexpression of GA20ox (Fig. 3 *E* and *F*). However, the predicted distribution of the nlsGPS1 emission ratio disagreed with that observed, as the model predicted overexpression of GA3ox would result in a substantial increase in GA_4_ in the meristem (Fig. 3 *B* and *F*). This discrepancy in the meristem was also present when comparing predicted and observed distributions for lines with both GA20ox and GA3ox overexpressed (Fig. 3 *C* and *G*) and lines with *ga20ox* loss-of-function (Fig. 3 *D* and *H*).

Given we observed no significant increase in GA_4_ levels in the central meristematic zone during the simultaneous induction of GA20ox and GA3ox expression (Fig. 3*C*), we hypothesized that these biosynthesis enzymes could be posttranscriptionally downregulated. Testing this hypothesis with the model, we simulated four cases, with reduced activity of GA3ox and GA20ox either i) throughout the meristem (*SI Appendix*, Fig. S8 *E*–*H* and *M*–*P*) or ii) in the division zone only (Fig. 3*I*–*L* and *SI Appendix*, Fig. S8 *I*–*L*), considering the GA3ox and GA20ox activities to be either reduced to zero (Fig. 3*I*–*L* and *SI Appendix*, Fig. S8 *E*–*H*) or reduced to 10% of their original value (*SI Appendix*, Fig. S8 *I*–*P*). Additionally, we reperformed the parameter survey to identify synthesis, delivery, and degradation rates for which the wild-type predictions align with the nlsGPS1 data (*SI Appendix*, Fig. S9 and Table S5).

We found that the model predictions are most consistent with the data provided GA20ox and GA3ox activity are set to zero in the DZ only (Fig. 3*I*–*L*; see *SI Appendix*, Table S6 for quantification of the Mean-Squared-Error (MSE) that characterizes the difference between the model predictions and data). All four cases substantially improved the agreement between predictions and data in wild type and the GA3ox overexpression line, as the reductions in GA20ox and GA3ox activity enabled a steeper GA_4_ gradient within the meristem (Fig. 3 *B* and *J* and *SI Appendix*, Fig. S8 *F*, *J*, and *N* and Table S6). Setting GA20ox and GA3ox activity to be zero in the DZ also improved the agreement between predictions and data for double overexpression of GA20ox and GA3ox, although these model predictions underestimated the increase in GA_4_ levels at the QCZ (Fig. 3 *C* and *K* and *SI Appendix*, Table S6). Predictions which also included zero or reduced activity in the QCZ would not capture this observed local increase of the GA_4_ levels in the QCZ in the GA20ox-GA3ox overexpression line, and the quantified MSE is larger for these cases (*SI Appendix*, Fig. S8 *G* and *O* and Table S6). In each of the four cases, predictions remained consistent with the observation that overexpressing GA20ox had minimal influence on the GA_4_ distribution (albeit with an increase in the quantified MSE compared to the original model assumptions) (Fig. 3 *A* and *I* and *SI Appendix*, Fig. S8 *E*, *I*, and *M* and Table S6). Similarly, in all four cases, model predictions reproduced the observations that loss-of-function mutations in either *ga20ox* or *ga3ox* resulted in a shallower GA_4_ gradient leading to lower GA_4_ levels (Fig. 3 *D* and *L* and *SI Appendix*, Fig. S8 *H*, *L*, and *P*).

**Fig. 4.**
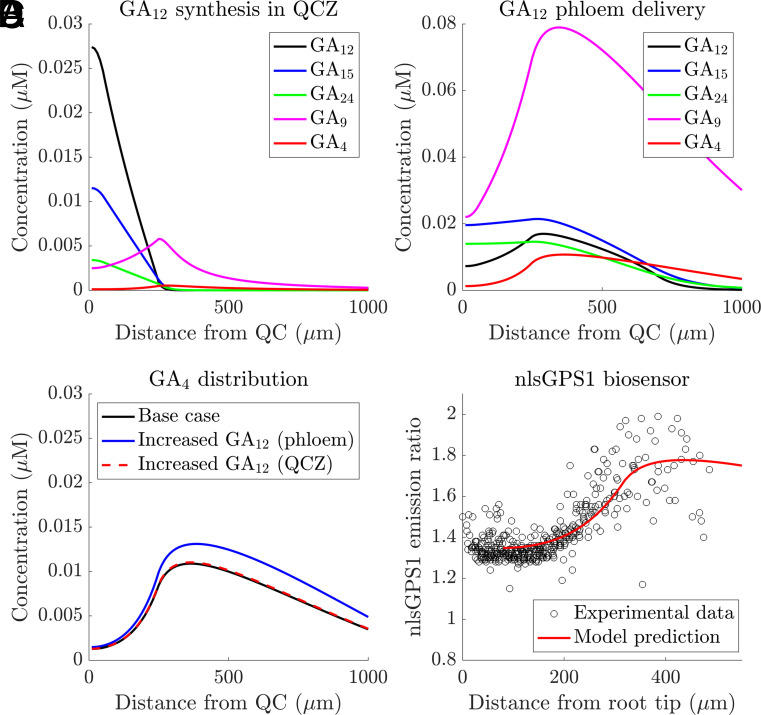
Model predictions with inactive GA20ox and GA3ox in the DZ reproduce GA_4_ gradient observed using the nlsGPS1 sensor (*A* and *B*) Predicted GA_12_, GA_15_, GA_24_, GA_9_, and GA_4_ concentration distributions due to (*A*) GA_12_ synthesis in the QCZ and (*B*) GA_12_ delivery the phloem-unloading zone. (*C*) Predicted GA_4_ distribution (due to both GA_12_ delivery in the phloem-unloading zone), together with predictions in which either the GA_12_ synthesis rate in the QCZ is doubled, or the GA_12_ delivery rate in the phloem-unloading zone is doubled. (*D*) Comparison between predicted and observed distribution of nlsGPS1 emission ratios. Predicted sensor distribution is calculated from the base case GA_4_ distribution shown in panel (*C*). The experimental data are reproduced from figure 1F in ref. 5 giving nlsGPS1 emission ratios from nuclei between 0 to 500 μm from the root tip. Quantification of the difference between the predictions and data, Mean-Squared-Error, MSE=0.0124 (*SI Appendix*, section 7).

Although setting GA20ox and GA3ox activity to be zero in the DZ was motivated by the data from the overexpression lines, the agreement between the predictions and data for wild type is improved substantially with this assumption (Fig. 4*D*). With inactive biosynthesis enzymes in the DZ, GA_12_ is able to diffuse further. As a result, the GA_12_ synthesized in the QCZ leads to GA_4_ synthesis in the EZ, and hence, both local GA_12_ synthesis in the QCZ and GA_12_ delivery via the phloem contribute to the GA_4_ gradient (Fig. 4*A*–*C*). However, the model predicts that GA_12_ delivery remains the dominant contributor to the GA_4_ levels, and increasing the rate of GA_12_ phloem delivery is predicted to have a more substantial effect than increasing the rate of local GA_12_ synthesis in the QCZ (Fig. 4*C*). With inactive biosynthesis enzymes in the DZ, the model predicts that the spatial distribution of GA_12_ synthesis/delivery has a lesser effect on the GA_4_ gradient: With uniform GA_12_ synthesis/delivery, the model predicts a GA_4_ gradient which is close to that observed via the nlsGPS1 sensor (*SI Appendix*, Fig. S10).

In conclusion, our results suggest that posttranscriptional regulation reduces the activity of GA20ox and GA3ox in the dividing cells. Such enzyme inactivation enables the model to reproduce the observed GA_4_ gradient in the GA3ox OE line, and improves agreement between predictions and data in the wildtype, *ga3ox*, *ga20ox*, and GA20ox-GA3ox overexpression lines.

### Plasmodesmatal Diffusion Refines the GA_4_ Gradient.

**Fig. 5.**
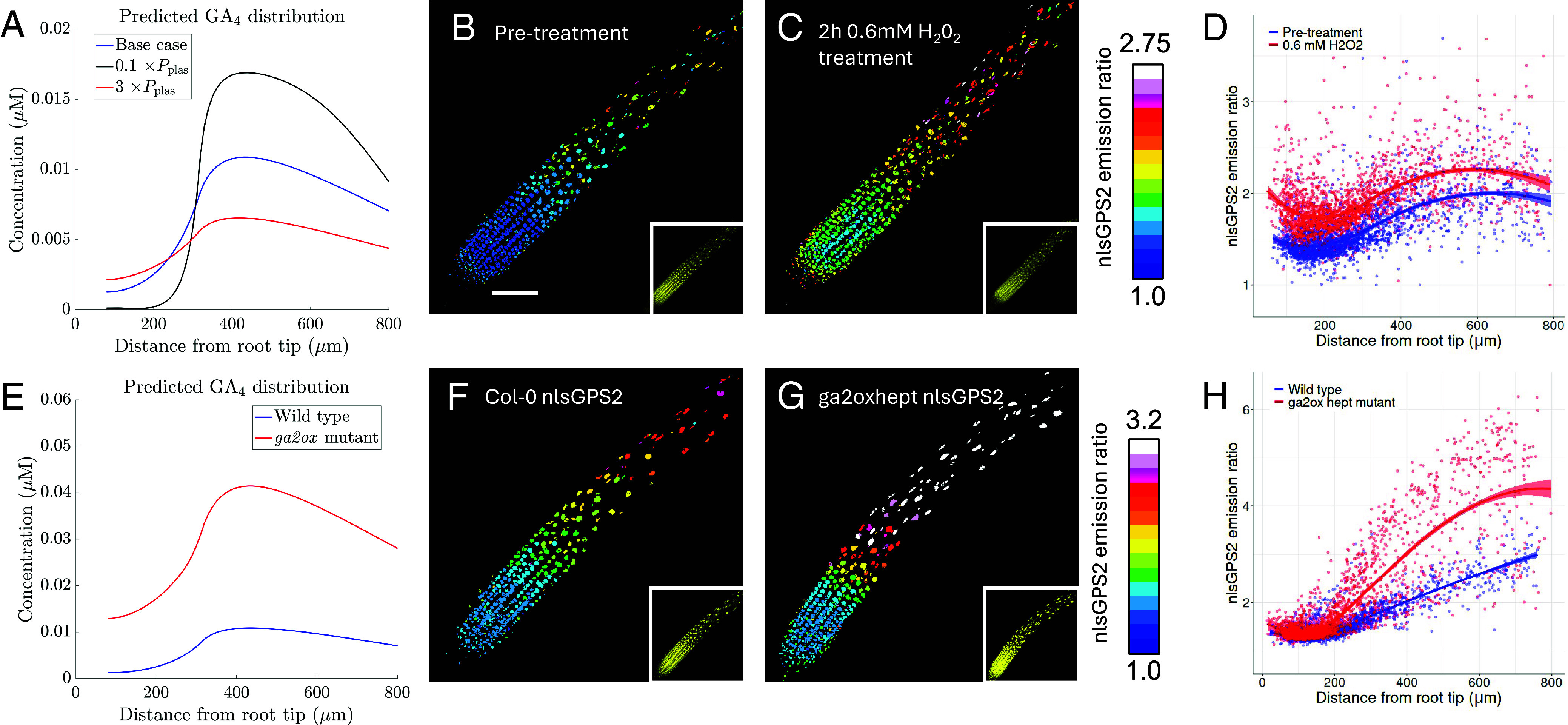
The GA_4_ gradient depends on plasmodesmatal diffusion and catabolism. (*A*) Predicted effect of the plasmodesmatal permeability, $P_{\mathrm{plas}}$, on the GA_4_ distribution. (*B* and *C*) Representative images of nlsGPS2 emission ratios and YFP fluorescence (*Inset*) for pretreated (*B*) and 2h 0.6mM H_2_O_2_ treated (*C*) roots. Treatment used to show the effect of increasing plasmodesmata permeability. (Scale bar, 100 μm applies to all images.) (*D*) Quantification of nlsGPS2 emission ratio data for pretreated and 2h 0.6mM H_2_O_2_ treated roots. Data quantified from images shown in panels (*B* and *C*), and *SI Appendix*, Fig. S12. (*E*) Predicted GA_4_ distribution for the *ga2ox* heptuple mutant (with reduced degradation of GA_12_, GA_9_ and GA_4_), (*F* and *G*) Representative images of nlsGPS2 emission ratios and YFP fluorescence (*Inset*) for Col0 (*F*) and *ga2oxhept* mutant (*G*) roots. (*H*) Quantification of nlsGPS2 emission ratio data for the *ga2ox* hept mutant. Data quantified from images shown in panels (*F* and *G*), and *SI Appendix*, Fig. S16. Panels (*D* and *H*) also show curves of best fit and 95% CIs for each dataset computed in R using local polynomial regression (Loess) via ggplot, with smoothing parameter span = 0.75 (as in ref. 5).

Plasmodesmatal diffusion has been shown to have a significant effect on the distributions of other hormones (11), although its role in GA_4_ distribution has not been explored. The model predicts that plasmodesmatal diffusion has a significant effect on the GA_4_ distribution: Reducing/increasing the plasmodesmatal permeability increases/decreases the slope of the GA_4_ gradient (Fig. 5*A* and *SI Appendix*, Fig. S11). With low plasmodesmatal diffusion, GA_12_ is unable to diffuse away from its synthesis/delivery regions (*SI Appendix*, Fig. S11*A*), and the downstream metabolites are also unable to diffuse leading to a steep GA_4_ gradient as cells enter the EZ (Fig. 5*A*). In contrast, with higher plasmodesmatal diffusion, the model predicts that the metabolites diffuse easily into the DZ, and the slope of the GA_4_ gradient is substantially reduced (Fig. 5*A*).

To test the model predictions that plasmodesmatal diffusion affects GA_4_ distribution, we experimentally perturbed plasmodesmatal permeability using a H_2_O_2_ treatment, choosing a treatment time of _2_ h and concentration of 0.6 mM which has previously been shown to increase plasmodesmatal permeabilities by approximately 3 times within the root tissues (33). Experiments were performed using the nlsGPS2 biosensor, which exhibits improved orthogonality and reversibility that is ideal for examining GA_4_ levels and in conjunction with growth phenotypes (35). Applying a 0.6 mM H_2_O_2_ treatment led to a shallower GA_4_ gradient (Fig. 5*B*–*D* and *SI Appendix*, Fig. S12) in agreement with the model predictions (Fig. 5*A*).

Interestingly, the H_2_O_2_ treatment was also observed to lead to a small increase in GA_4_ levels within the EZ (Fig. 5*D*). Given phloem unloading also occurs through plasmodesmata (27), one possible explanation for the observed increase in GA_4_ in the EZ is an increase in GA_12_ delivery via the phloem. Using the model to test this hypothesis, we found that small increases in the GA_12_ delivery rate are predicted to increase GA_4_ levels, as expected, although larger increases in delivery rate have limited effect, due to saturation of the biosynthesis steps (*SI Appendix*, Fig. S13). Combining an increase in GA_12_ delivery with an increased plasmodesmatal permeability still led to GA_4_ levels in the EZ that were lower than the pretreated case (*SI Appendix*, Fig. S14*A*), in contrast to our observations (Fig. 5*D*). We also explored the possibility is that the H_2_O_2_ treatment may be having a higher impact in the EZ, where the root is thought to be more permeable to external treatments (5), although the model again did not predict the observed higher GA_4_ in the EZ (*SI Appendix*, Fig. S14*B*), suggesting an additional factor is contributing to this GA_4_ increase under H_2_O_2_.

We conclude that appropriate plasmodesmatal diffusion is essential for shaping the GA_4_ gradient—if the plasmodesmatal diffusion is too high, the gradient will be eliminated, whereas if the diffusion is too low, the gradient would be much steeper than that observed.

### Dilution and NPF-Mediated Transport Have Limited Effect on the Predicted GA_4_ Distribution.

Since the cells grow, this naturally introduces dilution along the root, which would reduce concentrations. The model predicts that reducing dilution (by reducing the value of the cells’ RER) has a little effect on GA_4_ levels in the meristem and a small increase in GA_4_ levels in the EZ where the cell elongation is most rapid (*SI Appendix*, Fig. S15*A*).

Considering the NPF-mediated membrane transport, the model predicts a very small increase in GA_4_ in the EZ with vacuolar import removed (as in a *npf2.14* loss-of-function mutant), and little change in the meristem (*SI Appendix*, Fig. S15*B*). Since cell elongation in the EZ is predominantly due to vacuolar expansion, removing vacuolar import reduces dilution, explaining why these predictions are similar to those with reduced RER. The model predicts that removing the NPF cytoplasmic importers (such as in a *npf3.1* mutant) has no noticeable effect on the GA_4_ gradient (*SI Appendix*, Fig. S15*B*). These predictions suggest that the presence of NPF2.14 and NPF3.1 in particular tissues have little effect on the longitudinal GA_4_ gradient and thus suggest that the predictions are relevant to tissues both with and without these transporters.

We conclude that, while dilution and NPF2.14-mediated vacuolar import are predicted to have a small effect on the GA_4_ level in the EZ, plasmodesmatal diffusion is the dominant transport mechanism that controls the GA_4_ gradient.

### Catabolism Has Significant Effect on GA_4_ Levels.

We explored the effect of catabolism on GA_4_ distribution. Considering the heptuple *ga2ox* loss-of-function mutant (35), which has reduced catabolism of GA_12_, GA_9_ and GA_4_, the model predicts GA_4_ levels are higher than the wild type throughout the growth zones (Fig. 5*E*). To test this prediction, we examined nlsGPS2 biosensor emission ratios in the *ga2ox* heptuple mutant (35). The nlsGPS2 data revealed higher GA_4_ levels in the *ga2ox* heptuple mutant providing support for the model prediction (Fig. 5*F*–*H* and *SI Appendix*, Fig. S16).

Interestingly, the nlsGPS2 data suggested that the loss of degradation in the heptuple *ga2ox* mutant had a greater effect on the GA_4_ level in the EZ than in the meristem, suggesting that the GA2ox are particularly functional in the EZ. We note that the model predicts that reduced degradation also leads to higher GA_4_ in the meristem, in contrast to the *ga2oxhept* data (compare Fig. 5 *E* and *H*); this discrepancy could suggest there are other catabolic enzymes, for example, the ELAs, which are mediating GA_4_ degradation in the meristem.

Increasing the individual degradation rates (associated with GA_12_, GA_9_ or GA_4_) is predicted to lower the GA_4_ level (*SI Appendix*, Fig. S17). Consistent with this prediction, Kubalova et al. showed overexpression of GA2ox8 in the nlsGPS2 background resulted in lower GA_4_ levels (*Kubalovaet al., 2025 companion manuscript*). Increasing either the GA_4_ or GA_9_ degradation rates is predicted to result in a more substantial reduction of GA_4_ levels than increasing the GA_12_ degradation rate (*SI Appendix*, Fig. S17), although we note that increasing GA_12_ degradation may also reduce GA_12_ delivery from the shoot, which would further reduce the predicted GA_4_ levels.

We conclude that GA_4_ catabolism plays a key role in controlling GA_4_ levels and is essential to the GA_4_ gradient.

## Discussion

GA_4_’s role in plant growth regulation is well established (3). Our previous studies discovered a GA_4_ gradient in the *Arabidopsis* root growth zones that appears to underlie root cell elongation (4, 5). By developing a detailed multiscale mathematical model, we now analyze how cell-scale processes combine to create this GA_4_ gradient, via the combination of GA_12_ synthesis and delivery, spatial variations in biosynthesis enzyme activity, plasmodesmatal diffusion, and catabolism.

The model predicted that inactivation of the GA20ox and GA3ox biosynthesis enzymes in the DZ is essential to the GA_4_ gradient. We had previously observed that overexpressing the GA20ox and GA3ox enzymes did not increase GA_4_ levels in the DZ (5); comparing model predictions with these previous data suggested that these enzymes are inactive in the DZ. Furthermore, we found that enzyme inactivation refined the predicted wild-type gradient, leading to better agreement between predictions and data. This finding is aligned with that in Maize leaves that suggested posttranscriptional regulation to be important in regulating GA_1_ levels in response to drought and cold (34), where GA_1_ plays a similar role in regulating meristem size.

Our results suggest that plasmodesmatal diffusion antagonizes the GA_4_ gradient. Plasmodesmatal diffusion has been shown to affect distributions of auxin (11), but its influence on other hormone distributions was yet to be evaluated. Given plasmodesmatal permeabilities are rapidly regulated by environmental conditions (36, 37), a resulting modification to the GA_4_ gradient may provide an additional mechanism for environmental regulation of root growth. The presented model assumes that the plasmodesmatal permeability is constant; however, it is plausible that the plasmodesmatal permeability reduces as cells transition through the growth zones, potentially due to callose deposition (36, 37) or increased cell-wall thickness (38). Simulations suggest that a reduced plasmodesmatal permeability in the EZ would result in a steeper GA_4_ gradient (*SI Appendix*, Fig. S18). Detailed experimental measurements of plasmodesmatal permeabilities in different tissues, zones, and environmental conditions would greatly benefit such model developments and deepen our understanding of how plasmodesmata contribute to hormone gradients and root growth regulation. Hormone unloading from the phloem also occurs through plasmodesmata. The presented model assumes spatially uniform delivery of GA_12_; however, coupling it with a model of hormone advection within the phloem (such as ref. 39) would enable us to investigate how the phloem dynamics may lead to spatial variations in the GA_12_ delivery rate.

In contrast to the role of plasmodesmatal diffusion, the model predicted that the NPF-mediated active influx across the cell membranes has limited effect on the GA_4_ distribution. Given cytoplasmic GA_4_ is predominantly anionic and unable to cross membranes via passive diffusion (29), we predicted that the apoplastic GA_4_ concentrations are low and there is little GA_4_ available for influx via the plasma membrane carriers (NPF3.1, NPF2.12). Should GA_4_ efflux carriers be identified in the future, we anticipate that, where efflux occurs, apoplastic GA_4_ levels would be higher and influx carriers would then be predicted to contribute to the GA_4_ distribution. Plasma membrane import may also have an important role with exogenous gibberellin, for example NPF transporters were shown to affect the distribution of fluorescently tagged gibberellin between different tissues in the root cross-section (9, 10).

The model predictions suggest that phloem delivery of GA_12_ provides an important source of precursor to create high GA_4_ levels as cells enter the elongation zone. However, this does not rule out the possibility that local GA_12_ synthesis also plays an important role: Sensor emission ratios suggest a small peak in GA_4_ levels close to the QC, which may be derived from locally synthesized GA_12_ in this region. The strong GA_4_ in the QCZ with overexpression of both GA3ox and GA20ox also indicates a local pool of GA_12_ (5). A recent study that found inducing GA_4_ degradation specifically in the QCZ reduced meristem length, whereas inducing GA_4_ degradation in the shootward-meristem and EZ affected elongated cell length but not meristem length (40). If the QCZ and EZ GA_4_ pools do indeed have distinct functions and can be regulated independently, roots could use GA_4_ distribution to separately influence meristem size and cell elongation.

The model provides an explanation for the perturbed GA_4_ distributions observed in several characterized mutants. We predicted GA_4_ distributions for mutation and overexpression of GA2ox enzymes that have been corroborated experimentally in nlsGPS2 lines in *ga2ox* heptuple and GA2ox8 overexpression backgrounds (Fig. 5, and Kubalova et al., 2025, companion manuscript). The increased and reduced growth phenotypes of *ga2ox* heptuple and GA2ox8 overexpression lines evidence the quantitative relationship between cellular GA distribution and localized root growth (Kubalova, 2025 companion manuscript).

There have been numerous studies of plant hormone distributions focused on the hormone auxin, investigating how the auxin distributions are created, and how they mediate developmental processes and environmental responses (41). In comparison, our understanding of the distributions of other plant hormones is in relative infancy. Recently developed FRET sensors have revealed that other hormones, such as GA and ABA, also have dynamic spatial distributions, that are affected by genetic mutation and environmental conditions (4, 5, 35, 42–44). As in the auxin field (45), computational models, like that developed here, will be vital to explain and understand these hormone distributions and how they arise through processes at the cellular scale. We anticipate the model developed here will provide a basis for future modeling studies, incorporating additional processes such as hormone cross-talk.

Our study focuses on analyzing longitudinal GA_4_ gradients and develops a one-dimensional model to investigate GA_4_ distribution along a cell file. Comparing model predictions from biosensor data generated quantitative predictions regarding how GA_4_ gradients are formed and maintained in the root growth zones. The developed one-dimensional model retains some discrepancy with the biosensor data in the shootward region of the EZ where the model, based on transcription data (Fig. 1*D*), predicts lowering GA_4_ levels due to the combination of low biosynthesis and high catabolism (Fig. 5 *A* and *E*) while the nlsGPS2 biosensor reports that GA_4_ levels plateau (Fig. 5 *D* and *H*). More detailed biosensor imaging in the EZ and maturation zone would provide additional insights into the GA_4_ distribution in this region. Another limitation is that this one-dimensional approach is naturally unable to account for radial processes, such as plasmodesmatal diffusion between adjacent tissues; however, it paves the way for future three-dimensional models that include the multiple tissues in the root cross-section. Such three-dimensional models could predict how transport and plasmodesmatal diffusion between the tissue layers affect GA_4_ gradients and could more accurately represent phloem delivery; these radial processes were recently considered in a 2D multicellular model of the root cross-section, which suggested they may lead to high GA_4_ levels in the endodermis (10). We further note that the model here predicts GA_4_ distribution based on prescribed cell growth dynamics (using experimental measurements); developing a fully coupled model that incorporates hormone regulation of cell growth or division rates could be an interesting further extension.

We conclude that GA_4_ distributes like a classical morphogen, as proposed by Wolpert (12), with localized synthesis combining with diffusion and degradation to create a spatial gradient. At the same time, GA_4_ does not alone control the strict longitudinal patterning of QCZ, meristem, and EZ of the *Arabidopsis* root. Interestingly, the results presented in the companion manuscript show that auxin regulation of GA_4_ catabolism mediates growth responses (Kubalova et al., 2025). These findings could point to the GA_4_ gradient being a hub for other hormone signals, with regulation of our model inputs enabling other signals to regulate growth. The clear roles of GA_4_ in contributing to the root growth parameters suggest that other developmental pattern generators likely direct the GA_4_ enzymes and transport regimes uncovered here.

## Materials and Methods

### Mathematical Modeling.

Full details of the derivation of the model equations are provided in *SI Appendix*, sections 1–4. Where available, model parameter estimates are obtained from the literature (*SI Appendix*, Tables S1–S3), and the remaining five parameter values are estimated by comparing predictions with nlsGPS1 data (*SI Appendix*, section 6 and Table S4). Simulations were performed using matlab. All code and data used in this paper have been deposited in GitLab (https://gitlab.com/leahband/ga_arabidopsisroot_model/).

### Plant Material and Growth Conditions.

WT and mutant lines used in this study were *A. thaliana* ecotype Columbia 0 (Col-0). Seeds were chlorine-gas-sterilized and plated on 1/2 Murashige and Skoog (MS) basal medium (Duchefa, Cat No. M0221) with 0.025% 2-morpholinoethanesulfonic acid monohydrate (MES), pH 5.7, and 1.2% agar (1/2 MS solid medium, Sigma). After stratification in the dark at 4 °C for 3 d, plates were placed in a growth chamber with long-day growth conditions (120 μmol m^−2^ s^−1^ white light, 22 °C for 16 h; 0 μmol m^−2^ s^−1^, 18 °C for 8 h). Roots were imaged at 6 d postsowing with growth and cell zones at steady state. Lines used in this study nlsGPS2 for both Col-0 and *ga2oxhept* (35).

### Confocal Imaging and Treatments.

For steady-state experiments, samples were mounted in liquid 1/4 × MS medium (1/4 × MS salts, 0.025% MES, and pH 5.7) with coverslips and imaged. For the 0.6 mM H_2_0_2_ treatment, the standard medium beneath the coverslip was exchanged with the medium containing H_2_0_2_ solution. The H_2_0_2_ medium was prepared immediately before adding to the sample. A control buffer exchange was also carried out. The samples were then reimaged after 2 h. Confocal images were acquired with a format of 1,024 × 512 pixels and resolution of 12 bit on an upright Leica SP8 using a 20× dry 0.70 HC PLAN APO objective. To excite Cerulean and Aphrodite, 448 nm and 514 nm lasers were used, respectively. Emission filters were 460 to 500 nm for Cerulean and 525 to 560 nm for Aphrodite. Three fluorescence channels were collected for FRET imaging: Cerulean donor excitation and emission or DxDm, Cerulean donor excitation, Aphrodite acceptor emission or DxAm, and Aphrodite acceptor excitation and emission or AxAm.

### Image Processing and Analysis.

Imaging process and analysis were performed with FRETENATOR plugins (46). Segmentation settings were optimized for each experiment but kept constant within each experiment. The AxAm channel was used for segmentation. For segmentation, Otsu thresholds were used, difference of Gaussian kernel size was determined empirically, and a minimum ROI size was set to 20. Distance from meristem was defined using FRETENATOR ROI labeler.

## Supplementary Material

Appendix 01 (PDF)

## Data Availability

Computer code, image data, and Excel sheets of image quantification have been deposited in Code and data for “A balance of metabolism and diffusion articulates a gibberellin hormone gradient in the *Arabidopsis* root” (https://gitlab.com/leahband/ga_arabidopsisroot_model/) (47). Previously published data were used for this work (https://doi.org/10.17863/CAM.58366) (48).
