# Supplementary material for: A balance of metabolism and diffusion articulates a gibberellin hormone gradient in the Arabidopsis root
Source: Proc Natl Acad Sci U S A. 2025 Nov 24;122(48):e2425320122. doi: 10.1073/pnas.2425320122 (PMC12684920; doi:10.1073/pnas.2425320122)
Supplement: Supplementary file 1 — Appendix 01 (PDF) [file pnas.2425320122.sapp.pdf]

PNAS

Supporting Information for

A balance of metabolism and diffusion articulates a gibberellin hormone gradient in the *Arabidopsis* root

This PDF file includes:

Supporting text
Figs. S1 to S20
Tables S1 to S6
SI References

Supporting Information Text

In this Supplementary Information, we provide further details on the mathematical modelling.

We begin by describing the cell-based model for transport of a single hormone in a generic file of growing and dividing cells with constant hormone synthesis and degradation rates. We then use the results in (1) to derive a coarse-grained continuum model for the hormone concentration. In Section 1, we present the cell-based and continuum models incorporating the hormone transport dynamics. In Section 2, we discuss the root growth dynamics and the corresponding assumptions used in the model. In Section 3, we verify that the continuum approximation recapitulates the output of the cell-based model using the growth dynamics described in Section 2. In Section 4, we describe the model of the gibberellin metabolic pathway (as shown in Fig. 1B in the main text). In Section 5, we describe the full model that incorporates transport dynamics, growth, and the metabolic pathway, detailed in Sections 1–4. In Section 6, we describe how parameter estimates were obtained: the majority of the model parameter values are obtained from the literature (as listed in Tables S1-S3); however, five parameter values are unavailable and were estimated by comparing model predictions with the wildtype nlsGPS1 data, as detailed in section 6 and given in Table S5.

1. Modelling hormone transport via a continuum approximation of a cell-based model

Before presenting the full model, we first describe hormone transport in a model for a single hormone to demonstrate how a cell-based transport model can be approximated by a continuum equation. We consider transport in a file of length $L(t)$ of N growing and dividing cells with apoplastic (cell wall) and subcellular (vacuolar) compartments, as shown in Supp. Fig. S2. We incorporate passive transport and facilitated transport via protein transporters located on the cytoplasmic and vacuolar membranes of the cells. We include plasmodesmatal transport between cell cytoplasms and apoplastic diffusion within the cell wall. We use the notation and model description in (1) to express the rate of change of hormone concentration in each compartment in terms of the net flux of the hormone through the relevant boundaries. Further, we assume that hormone is produced at a rate σ and degraded at a rate β within each cytoplasm (noting that these terms will be replaced in the full model described in Section 5). Initially, there is no hormone in the cells. The hormone dynamics can be modelled via a system of coupled ordinary differential equations for the hormone concentrations in the cytoplasm (c_i), vacuole (v_i), and adjacent apoplast compartments (f_i, g_i, and h_i), where, as in (1), we assume that the hormone concentrations are constant within each compartment:

$$w\frac{\mathrm{d}}{\mathrm{d}t}\left((1-\phi_i)l_ic_i\right) = w(J_{fci} - J_{cfi} + J_{cci} - J_{cc(i-1)}) - l_iJ_{chi} - 2\sqrt{\phi_i}(l_i+w)J_{cvi} + w(1-\phi_i)l_i(\sigma - \beta c_i), \quad [1a]$$

$$w\frac{\mathrm{d}}{\mathrm{d}t}\left(\phi_il_iv_i\right) = 2\sqrt{\phi_i}(l_i+w)J_{cvi}, \quad [1b]$$

$$aw\frac{\mathrm{d}f_i}{\mathrm{d}t} = w(J_{cfi} - J_{fc(i+1)}) - aJ_{fgi}, \quad [1c]$$

$$a^2\frac{\mathrm{d}g_i}{\mathrm{d}t} = a(J_{hgi} - J_{gh(i+1)} + J_{fgi}), \quad [1d]$$

$$a\frac{\mathrm{d}}{\mathrm{d}t}\left(l_ih_i\right) = l_iJ_{chi} + a(J_{ghi} - J_{hgi}), \quad [1e]$$

which hold for $2 \leq i \leq N$, where w is the width of the cells, a is the thickness of the apoplastic compartments, l_i are the cell lengths, ϕ_i are the vacuolar area fractions in each cell, and J_{xyi} is the flux from compartment

x to compartment y in cell i, i.e.,

$$J_{cfi} = P_{\mathrm{ca}}c_i - P_{\mathrm{ac}}f_i, \qquad [2a]$$

$$J_{cvi} = P_{\mathrm{cv}}c_i - P_{\mathrm{vc}}v_i, \qquad [2b]$$

$$J_{fci} = P_{\mathrm{ac}}f_{i-1} - P_{\mathrm{ca}}c_i, \qquad [2c]$$

$$J_{chi} = P_{\mathrm{ca}}c_i - P_{\mathrm{ac}}h_i, \qquad [2d]$$

$$J_{fgi} = \frac{2D_{\mathrm{apo}}}{w+a}(f_i - g_i), \qquad [2e]$$

$$J_{ghi} = \frac{2D_{\mathrm{apo}}}{l_i+a}(g_{i-1} - h_i), \qquad [2f]$$

$$J_{hgi} = \frac{2D_{\mathrm{apo}}}{l_i+a}(h_i - g_i), \qquad [2g]$$

$$J_{cci} = P_{\mathrm{plas}}(c_{i+1} - c_i). \qquad [2h]$$

Descriptions of the remaining parameters can be found in Supplementary Tables S1 and S2, and further explanation is provided in (1).

We assume the hormone cannot leave the file at either end, and thus, the governing equations Eq. (1) are subject to the following boundary conditions

$$c_1 = c_2, \qquad h_1 = h_2, \qquad [3a]$$

$$c_N = c_{N-1}, \qquad h_N = h_{N-1}, \qquad [3b]$$

and initial conditions

$$c_i = v_i = f_i = g_i = h_i = 0 \qquad \text{at} \qquad t = 0 \qquad \text{for} \quad 1 \le i \le N. \qquad [4]$$

We note that, in contrast to (1), here, we have additional production and degradation terms in equation Eq. (1a), whereas Eq. (3) are no-flux (Neumann), rather than a Dirichlet condition.

Following the analysis in (1), we use Eq. (1)–Eq. (4) to derive a continuum description of the system, where all variables (hormone concentrations, cell lengths, and vacuolar fractions) become functions of space, x, along the file and time, t, e.g., $c_i \approx C(x,t)$, $l_i(t) \approx l(x,t)$, etc. This approach greatly simplifies the computational challenges associated with solving for the discrete model in every cell accounting for cell growth and division. Additionally, through the derivation of effective course-grained parameters, such as effective diffusivity and advective velocity, it provides insight into what the effect of each parameter on the system behaviour is.

Exploiting the fact that the cell lengths are much less than the tissue length, it can be shown that the hormone concentrations in the apoplastic and vacuolar compartments are approximately proportional to the cytoplasmic concentration (see (1)), i.e.,

$$f = g = h = \mathcal{P}_{\mathrm{a}}C, \qquad v = \mathcal{P}_{\mathrm{v}}C, \qquad [5]$$

where

$$\mathcal{P}_{\mathrm{a}} = P_{\mathrm{ca}}/P_{\mathrm{ac}}, \qquad \mathcal{P}_{\mathrm{v}} = P_{\mathrm{cv}}/P_{\mathrm{vc}}. \qquad [6]$$

With this, following the derivation in (1), we can show that the discrete model Eq. (1) can be approximated by a continuum reaction–advection–diffusion equation for the cytoplasmic concentration $C(x,t)$. This takes the following form

$$\frac{\partial C}{\partial t} + \frac{\partial}{\partial x}\left(U_{\mathrm{eff}}(x,t)C\right) = \frac{\partial}{\partial x}\left(D_{\mathrm{eff}}(x,t)\frac{\partial C}{\partial x}\right) - Q_{\mathrm{eff}}(x,t)C + \frac{(1-\phi)l(\sigma-\beta C)}{V}, \qquad [7]$$

where

$$U_{\mathrm{eff}}(x,t) = u + \frac{K(l+a)+M}{V}\frac{\partial l}{\partial x} - \frac{(K(l+a)+M)(l+a)}{V^2}\frac{\partial V}{\partial x}, \tag{8a}$$

$$D_{\mathrm{eff}}(x,t) = \frac{(K(l+a)+M)(l+a)}{V}, \tag{8b}$$

$$Q_{\mathrm{eff}}(x,t) = \frac{u}{V}\frac{\partial V}{\partial x} - \frac{\partial U_{\mathrm{eff}}}{\partial x} + \frac{1}{V}\frac{\partial V}{\partial t} = \frac{1}{V}\frac{\mathrm{d}V}{\mathrm{d}t} - \frac{\partial U_{\mathrm{eff}}}{\partial x}, \tag{8c}$$

and, for convenience, we have defined

$$\begin{gathered} K = wP_{\mathrm{ca}}/2 + wP_{\mathrm{plas}}, \quad M = D_{\mathrm{apo}}a\mathcal{P}_{\mathrm{a}}, \\ V = (1-\phi)wl + \phi wl\mathcal{P}_{\mathrm{v}} + a(w+a+l)\mathcal{P}_{\mathrm{a}}, \end{gathered} \tag{9}$$

where $u(x,t)$ is the advective velocity due to growth calculated from the growth rate, as in (1). In equation (7), the second term represents advection, with $U_{\mathrm{eff}}(x,t)$ being the effective advection rate, the third term represents diffusion, with $D_{\mathrm{eff}}(x,t)$ being the effective diffusivity, and the final two terms are the reaction terms, with the penultimate term representing dilution and $Q_{\mathrm{eff}}(x,t)$ being the effective dilution rate, and the final term representing production and degradation. From Eq. (3) and Eq. (4), equation (7) is subject to

$$\frac{\partial C}{\partial x} = 0 \quad \text{at} \quad x = 0, \tag{10a}$$

$$\frac{\partial C}{\partial x} = 0 \quad \text{at} \quad x(t) = L(t), \tag{10b}$$

$$C = 0 \quad \text{at} \quad t = 0. \tag{10c}$$

Eq. (7) subject to Eq. (10) is equivalent to the corresponding equation derived in (1), albeit with the addition of the final term in Eq. (7) that represents synthesis and degradation.

2. Modelling cell growth and division within the *Arabidopsis* root

A cell file in the *Arabidopsis* root comprises three different growth zones with their own specific cell dynamics. The first zone, adjacent to the root tip, is the meristem, where cells rapidly divide, and the cell elongation rate is slow. We assume every cell in the meristem divides once it approximately doubles in length. The next zone, adjacent to the meristem, is the elongation zone, where cells do not divide but grow rapidly. Here, cells increase their length almost 10-fold (2). The third zone, adjacent to the elongation zone, is the maturation zone, where cells have attained their final length and do not grow, nor divide. In the model, we simulated a portion of the maturation zone, and took the end of the file to be at position $x = L_{\mathrm{file}} = 1300\mu$m; we checked that using different values for L_{file} resulted in simulation outputs that looked identical to those with the $L_{\mathrm{file}} = 1300\mu$m used. We assume that the metabolite concentrations and their fluxes are continuous across the boundaries between the zones.

We focus on mature seedlings, in which the sizes of the meristem and the elongation zone are stable, and denoted by L_{meri} and L_{ez}, respectively. Thus, we assume the cell lengths evolve at a quasi-steady state, i.e., there is approximately the same number of cells in each zone, and the cell lengths and vacuolar fractions are only functions of distance away from the root tip. Using data in (3), we assume that the cell elongation rates in the meristem and the elongation zone are constant and equal to $\mathrm{RER}_{\mathrm{meri}}$ and $\mathrm{RER}_{\mathrm{ez}}$, respectively. Thus, for every cell i, its length evolves according to

$$\frac{\mathrm{d}l_i}{\mathrm{d}t} = \mathrm{RER}_{\mathrm{zone}}l_i, \tag{11}$$

where "zone" refers to either the meristem (meri) or the elongation zone (ez). Within the meristem, cells undergo division at discrete times, with the time between two successive cell divisions given by

$$T_{\mathrm{d}} = \frac{\ln(2)}{\mathrm{RER}_{\mathrm{meri}}}, \tag{12}$$

see (4).

In the meristem, cell growth is due to both cytoplasmic and vacuolar expansion (5). To maintain a constant vacuole volume fraction throughout the meristem, ϕ_{meri} (as observed in (5)), we must assume that the cytoplasm and the vacuole expand at the same rate, $\mathrm{RER}_{\mathrm{meri}}$. In the elongation zone, previous models (6, 7) had assumed that cell elongation is entirely due to vacuolar expansion; however, new data (5, 8) contradicts this previous assumption and reveals that the cytoplasm continues to expand slowly in the elongation zone. Calculations detailed in section 8 below show that these new data (5, 8) are consistent with the model assumptions that the cytoplasm expands slowly at the cell elongation rate in the meristem, while the vacuole expands more rapidly to enable the cells to elongate at a rate of $\mathrm{RER}_{\mathrm{ez}}$ (larger than $\mathrm{RER}_{\mathrm{meri}}$). Using Eq. (38) and Eq. (39), we obtain the following governing equation for ϕ_i in the EZ

$$\frac{1}{1-\phi_i}\frac{\mathrm{d}\phi_i}{\mathrm{d}t} = \mathrm{RER}_{\mathrm{ez}} - \mathrm{RER}_{\mathrm{meri}}. \qquad [13]$$

In the maturation zone, there is no cell elongation and therefore we assume the vacuolar volume fraction is constant, denoted ϕ_{mat}.

We now have enough information to solve the discrete model Eq. (1)–Eq. (4) with Eq. (11) and Eq. (13) to predict the distribution of a single gibberellin metabolite.

Having described the growth assumptions in terms of the cell-based model, we now need to determine the corresponding continuum growth model by deriving corresponding formulae for the average continuum distribution of cell lengths, $l(x,t)$, vacuolar fractions, $\phi(x,t)$, and cell velocity, $u(x,t)$.

In the meristem, data (3, 5, 6, 8) suggest that the average cell lengths and the vacuolar fractions are approximately spatially constant, denoted by l_{meri} and ϕ_{meri}, respectively. Due to the continual cell division, we take l_{meri} to be the average cell length during one cell cycle, i.e.,

$$l_{\mathrm{meri}} = \frac{1}{T_{\mathrm{d}}}\int_0^{T_{\mathrm{d}}} l_i \,\mathrm{d}t, \qquad [14]$$

where T_d is the time between two successive divisions, (Eq. (12)). We can find the relationship between the initial cell length, l_0, (immediately after the division event) and the average cell length, l_{meri}, by substituting Eq. (11) and Eq. (12) into Eq. (14), noting that the quasi-steady state assumption requires the cell length to double between two successive divisions; this gives us

$$l_{\mathrm{meri}} = \frac{l_0}{\ln(2)}. \qquad [15]$$

With a value of $l_0 = 6\,\mu$m from (2), we calculate the average cell length to be $l_{\mathrm{meri}} = 8.7\,\mu$m, which is consistent with the distributions of cell lengths shown in (2). Given that the relative elongation rates are taken to be constant within each zone, in the quasi-steady frame of reference, the cell lengths follow a linear profile in the elongation zone with respect to distance along the root, using Eq. (11) as explained in (6). In particular, the relation

$$\frac{\mathrm{d}l}{\mathrm{d}x} = c\mathrm{RER}_{\mathrm{ez}} \qquad [16]$$

holds, where the constant

$$c = \frac{l_{\mathrm{meri}}}{\mathrm{RER}_{\mathrm{meri}} L_{\mathrm{meri}}} \qquad [17]$$

is the average time between successive cells leaving the meristem, and hence entering/leaving the EZ (see (4, 6, 9–11)).

In the maturation zone, the average cell lengths and the vacuolar fractions are constant, denoted by l_{mat} and ϕ_{mat} respectively, which is consistent with data (3, 12). By integrating (16), we see that the average cell length in the maturation zone can be calculated via

$$l_{\mathrm{mat}} = l_{\mathrm{meri}} + c\mathrm{RER}_{\mathrm{ez}} L_{\mathrm{ez}}. \qquad [18]$$

The velocity is calculated using the elongation rates, as explained in (1), namely, using a mass conservation law for the cell density and expressing the gradient of the velocity as the RER in the corresponding zone. Therefore, we let

$$l(x) = \begin{cases} l_{\text{meri}} & \text{if } x \le L_{\text{meri}} \\ l_{\text{meri}} + (x - L_{\text{meri}})(l_{\text{mat}} - l_{\text{meri}})/L_{\text{ez}} & \text{if } L_{\text{meri}} < x \le L_{\text{meri}} + L_{\text{ez}} \\ l_{\text{mat}} & \text{if } x > L_{\text{meri}} + L_{\text{ez}} \end{cases},$$

$$\phi(x) = \begin{cases} \phi_{\text{meri}} & \text{if } x \le L_{\text{meri}} \\ \phi_{\text{meri}} + (x - L_{\text{meri}})(\phi_{\text{mat}} - \phi_{\text{meri}})/L_{\text{ez}} & \text{if } L_{\text{meri}} < x \le L_{\text{meri}} + L_{\text{ez}} \\ \phi_{\text{mat}} & \text{if } x > L_{\text{meri}} + L_{\text{ez}} \end{cases}, \quad [19]$$

$$u(x) = \begin{cases} \text{RER}_{\text{meri}} x & \text{if } x \le L_{\text{meri}} \\ \text{RER}_{\text{ez}}(x - L_{\text{meri}}) + \text{RER}_{\text{meri}} L_{\text{meri}} & \text{if } L_{\text{meri}} < x \le L_{\text{meri}} + L_{\text{ez}} \\ \text{RER}_{\text{ez}} L_{\text{ez}} + \text{RER}_{\text{meri}} L_{\text{meri}} & \text{if } x > L_{\text{meri}} + L_{\text{ez}} \end{cases}.$$

In Supp. Fig. S1, we show plots of the cell elongation rate, length, and velocity using equations Eq. (19) and the parameter values listed in Table S1.

3. Comparison between continuum and cell-based model

Before proceeding with modelling GA_4 metabolism, we first verified that predictions using the continuum approximation agree with those of the cell-based model using the growth dynamics detailed in Section 2. Given the continuum approximation of the transport model was already carefully verified in (1), we here focus on testing the derived growth dynamics, using a simpler transport model in which we omit the apoplast and assume GA_4 moves between compartments only via plasmodesmata. For concreteness, we set the spatial variations in synthesis rate using the form suggested in (6):

$$\sigma(x) = \sigma_{QC} + \frac{\alpha x^n}{\xi^n + x^n}, \quad [20]$$

with parameter values $\sigma_{QC} = 0.00005\mu\text{M/hr}$, $\alpha = 0.0006\mu\text{M /hr}$, $\xi = 125\mu\text{m}$ and $n = 10$. We used a constant degradation rate, $\beta = 50/\text{hr}$.

For the cell-based model, we solve the system of ODEs (1a,b) with (2h) setting $J_{cfi} = J_{fci} = J_{chi} = J_{fgi} = J_{ghi} = J_{hgi} = 0$. These equations are subject to boundary and initial conditions given by (3) and (4), and are coupled to the ODEs describing the growth dynamics, Eq. (11), and Eq. (13).

For the continuum model, we solve the PDE problem Eq. (7)–Eq. (10) with the growth dynamics as prescribed in Eq. (19). In solving the continuum model, we find it convenient to define the following zone-dependent formula using Eq. (9), in the meristem,

$$\frac{\text{d}V}{\text{d}t} = \text{RER}_{\text{meri}}((1 - \phi_{\text{meri}})w + \phi_{\text{meri}} w \mathcal{P}_{\text{v}} + a\mathcal{P}_{\text{a}})l, \quad [21]$$

and in the elongation zone,

$$\frac{\text{d}V}{\text{d}t} = \text{RER}_{\text{meri}}(1 - \phi)wl + (\text{RER}_{\text{ez}} - (1 - \phi)\text{RER}_{\text{meri}})wl\mathcal{P}_{\text{v}} + \text{RER}_{ez} al\mathcal{P}_{\text{a}}. \quad [22]$$

We use MATLAB *ode15s* package in both cases and run the simulations to large times to obtain the steady-state solutions. We show a comparison between the discrete and the continuum model in Supp. Fig. S3 and we see excellent agreement. Having now verified this agreement, we proceed to use the continuum formulation of the model to incorporate the metabolic pathway and transport of each gibberellin metabolite.

4. Modelling the GA_4 synthesis and degradation pathway focusing on metabolism downstream of GA_{12}

In order to study the biosynthesis of the bioactive form GA_4, we incorporate the metabolic pathway, whereby GA_4 is synthesised from GA_{12}. The GA_{12} is initially converted to GA_{15} under the action of the GA20ox enzymes. The GA20ox enzymes also convert GA_{15} to GA_{24} and convert GA_{24} to GA_9. Under the action of the GA3ox enzymes, GA_9 is converted into GA_4. Thus, GA_4 synthesis is mediated via the following steps:

$$\mathrm{GA}_{12} \xrightarrow{\mathrm{GA_{20ox}}} \mathrm{GA}_{15} \xrightarrow{\mathrm{GA_{20ox}}} \mathrm{GA}_{24} \xrightarrow{\mathrm{GA_{20ox}}} \mathrm{GA}_{9} \xrightarrow{\mathrm{GA_{3ox}}} \mathrm{GA}_{4}. \qquad [23]$$

Several of the GA metabolites are degraded via the GA2oxidase enzymes. Since different forms of GA2ox are involved in degradation of GA_{12}, GA_9, and GA_4, we denote them by GA2oxA, GA2oxB, and GA2oxC, respectively (13). Thus, GA_4 degradation is mediated via the following steps:

$$\mathrm{GA}_{12} \xrightarrow{\mathrm{GA_{2oxA}}} \text{degradation}, \qquad [24]$$

$$\mathrm{GA}_{9} \xrightarrow{\mathrm{GA_{2oxB}}} \text{degradation}, \qquad [25]$$

$$\mathrm{GA}_{4} \xrightarrow{\mathrm{GA_{2oxC}}} \text{degradation}. \qquad [26]$$

In the model, we assume that GA_{12} (i) can be produced within the quiescent centre region (QCZ), which we take to be the region within 43.5 μm of the QC (approximately the first five cells of the meristem), and (ii) can be delivered to cells within the phloem-unloading zone, which has been shown to be approximated by the most rootward half of the elongation zone (14). These two sources of GA_{12} will be referred to from now on as the QC and phloem GA_{12} pools.

5. Full model

In order to model the dynamics of all gibberellin metabolites, described in Section 4, we first extend the continuum model Eq. (7)–Eq. (10) from Section 1 to model transport and metabolism of each GA metabolite with the synthesis and degradation terms replaced with the corresponding terms from the metabolic pathway. Transport is accounted for through the diffusivity and the permeability values of the transporters and plasmodesmata, whereas cell growth and division is modelled as in Section 2 (see Eq. (11)). To overcome the increasing computational complexity due to the number of equations and evolving geometry involved we use the continuum approximation introduced at the end of Section 1. This leads to a system of five reaction–advection–diffusion equations for the concentration of each gibberellin metabolite. We model the biosynthesis steps by assuming Michaelis-Menten kinetics for the enzyme and metabolite concentrations participating in the corresponding reaction. In the formula for the GA20ox-mediated steps, we follow (15), noting that the formulas for these three reactions are more complicated than a single-step enzyme reaction, since GA20ox participates in all of them. GA2ox-mediated degradation is modelled via linear terms as in (16).

$$\frac{\partial[\mathrm{GA}_j]}{\partial t} + \frac{\partial}{\partial x}\left(U_{\mathrm{eff}}(x,t)[\mathrm{GA}_j]\right) = \frac{\partial}{\partial x}\left(D_{\mathrm{eff}}(x,t)\frac{\partial[\mathrm{GA}_j]}{\partial x}\right) - Q_{\mathrm{eff}}(x,t)[\mathrm{GA}_j] + S_j \quad \text{for} \quad j = 12, 15, 24, 9, 4, \qquad [27]$$

where

$$S_{12} = \frac{(1-\phi)wl\psi(x)}{V}\left(S(x) - \frac{\lambda_{12}[\mathrm{GA}_{12}][\mathrm{GA20ox}]}{1+\kappa_{12}[\mathrm{GA}_{12}]+\kappa_{15}[\mathrm{GA}_{15}]+\kappa_{24}[\mathrm{GA}_{24}]} - \beta_{12}[\mathrm{GA}_{12}][\mathrm{GA2oxA}]\right), \qquad [28a]$$

$$S_{15} = \frac{(1-\phi)wl\psi(x)(\lambda_{12}[\mathrm{GA}_{12}][\mathrm{GA20ox}] - \lambda_{15}[\mathrm{GA}_{15}][\mathrm{GA20ox}])}{V(1+\kappa_{12}[\mathrm{GA}_{12}]+\kappa_{15}[\mathrm{GA}_{15}]+\kappa_{24}[\mathrm{GA}_{24}])}, \qquad [28b]$$

$$S_{24} = \frac{(1-\phi)wl\psi(x)(\lambda_{15}[\mathrm{GA}_{15}][\mathrm{GA20ox}] - \lambda_{24}[\mathrm{GA}_{24}][\mathrm{GA20ox}])}{V(1+\kappa_{12}[\mathrm{GA}_{12}]+\kappa_{15}[\mathrm{GA}_{15}]+\kappa_{24}[\mathrm{GA}_{24}])}, \qquad [28c]$$

$$S_9 = \frac{(1-\phi)wl\psi(x)}{V}\left(\frac{\lambda_{24}[\mathrm{GA}_{24}][\mathrm{GA20ox}]}{1+\kappa_{12}[\mathrm{GA}_{12}]+\kappa_{15}[\mathrm{GA}_{15}]+\kappa_{24}[\mathrm{GA}_{24}]} - \frac{\lambda_9[\mathrm{GA}_9][\mathrm{GA3ox}]}{1+\kappa_9[\mathrm{GA}_9]} - \beta_9[\mathrm{GA}_9][\mathrm{GA2oxB}]\right), \quad [28d]$$

$$S_4 = \frac{(1-\phi)wl\psi(x)}{V}\left(\frac{\lambda_9[\mathrm{GA}_9][\mathrm{GA3ox}]}{1+\kappa_9[\mathrm{GA}_9]} - \beta_4[\mathrm{GA}_4][\mathrm{GA2oxC}]\right). \quad [28e]$$

Here, [GA20ox], [GA3ox], [GA2oxA],[GA2oxB],[GA2oxC] are the corresponding enzyme transcript levels, which are given functions of x (see Table S3), where, based on current understanding of the GA2ox family [GA2oxB] = [GA2oxC], $S(x)$ is the production/delivery rate of GA_{12} (from either the QC or phloem pool, see Eq. (32) below), D_{eff}, U_{eff}, and Q_{eff} are given in Eq. (8), l, ϕ, and u are given in Eq. (19), and the rate constants λ_j and κ_j, $j = 12, 5, 24, 9, 4$, are given in Table S1.

In order to simplify Eq. (27) to compute numerical solutions, we expand the derivatives to obtain the simplified equations

$$\frac{\partial[\mathrm{GA}_j]}{\partial t} = R(x)(l+a)\frac{\partial^2[\mathrm{GA}_j]}{\partial x^2} + \left(R(x)\frac{\partial l}{\partial x} - u\right)\frac{\partial[\mathrm{GA}_j]}{\partial x} - \frac{1}{V}\frac{\mathrm{d}V}{\mathrm{d}t}[\mathrm{GA}_j] + S_j \quad \text{for} \quad j = 12, 15, 24, 9, 4, \quad [29]$$

where

$$R(x) = \frac{(K(l+a)+M)}{V}, \quad [30]$$

and K, M, and V are given in Eq. (9).

Equations Eq. (29) with Eq. (28a)–Eq. (28e) are solved separately in the meristem, elongation zone, and maturation zone with Eq. (21) and Eq. (22), and are subject to the following boundary and initial conditions

$$\frac{\partial[\mathrm{GA}_j]}{\partial x} = 0 \quad \text{at} \quad x = 0, \quad [31a]$$

$$\frac{\partial[\mathrm{GA}_j]}{\partial x} = 0 \quad \text{at} \quad x = L_{\mathrm{file}}, \quad [31b]$$

$$[\mathrm{GA}_j] = 0 \quad \text{at} \quad t = 0, \quad [31c]$$

where $j = 12, 15, 24, 9, 4$. Additionally, each $[\mathrm{GA}_j]$ and $\partial[\mathrm{GA}_j]/\partial x$ are assumed to be continuous across the boundaries between the meristem, elongation zone, and the maturation zone, i.e., at $x = L_{\mathrm{meri}}$ and $x = L_{\mathrm{meri}} + L_{\mathrm{ez}}$. We solve the time-dependent equations for large time when transport dynamics has stabilised.

We define the production rate of GA_{12} to be

$$S(x) = \begin{cases} S_{\mathrm{QC}} & \text{if } 0 \le x \le L_{\mathrm{QC}} \\ S_{\mathrm{phloem}} & \text{if } L_{\mathrm{meri}} \le x \le L_{\mathrm{meri}} + L_{\mathrm{phloem}} \\ 0 & \text{otherwise} \end{cases}, \quad [32]$$

where L_{phloem} is the length of the phloem-unloading zone assumed to be half the length of the elongation zone, S_{QC} is the rate of GA_{12} production in the QCZ, and S_{phloem} is the rate of GA_{12} delivery in the phloem-unloading zone, respectively.

6. Parameter estimates

In Supplementary Tables S1-S3 and S5, we list the model parameters together with the parameter values used in the simulations presented in the main text.

In Supplementary Tables S1, we list the physical model parameters with estimates of their values used in the model. As described below, these values were obtained from the experimental literature, using values that pertain to the hormone gibberellin in the model species *Arabidopsis thaliana*.

The cell growth dynamics are prescribed using experimental data from the literature. The lengths of the meristem and elongation zones are prescribed using data generated in parallel with the sensor emission ratio data (6), relative elongation rates are as measured in (3), and the resulting cell length distribution is generated by prescribing the initial cell length as in the cortical cell data in (2). With this value of $l_0 = 6\,\mu$m (2), we calculate the average cell length to be $l_{meri} = 8.7\,\mu$m via Eq. (19). These parameter values generate the cell-length distribution shown in Figure S1B, which is consistent with the cell-length distributions measured in (2, 11). As stated in the main text, the sensor data are primarily emission ratios from the outer cell layers in the root growth zone. Due to the availability of cell growth data from the cortex, we parameterise the model using cortical cell measurements. We note that data in (11) suggests cell lengths in the epidermis are similar to those in the cortex, with the epidermal trichoblast cells having cell lengths almost identical to the cortical cells, whereas the atrichoblast cells have a slightly larger length. We tested the robustness of our results to the prescribed cell lengths, finding that prescribing a larger meristem cell length results in larger cell lengths throughout the growth zones and leads to a small reduction in the predicted GA_4 gradient (Fig. S19).

The model incorporates apoplastic diffusion, and we specify the metabolite diffusivity estimated in (17) and the apoplast thickness measured in (18); however, we note that simulations suggest that the value for the apoplastic thickness has little influence on the predicted GA_4 gradient (Fig. S20).

The plasmodesmatal permeabilities have been characterised in (19), with tissue-scale measurements suggesting a value of $8.5\,\mu$m/s in the inner tissue layers, and cell-scale measurements suggesting a value of $3.3\,\mu$m/s in the epidermis. However, as noted by (20), (21) report an approximately 2-fold difference in plasmodesmatal density on these cell-to-cell walls of the epidermis compared with those in the interior root tissues, providing a plausible explanation of the difference between these two permeability measurements. Given the sensor data is predominantly from the root's outer layers, we use the estimated epidermal value, $P_{plas} = 3.3\,\mu$m/s (19).

The membrane transport rates given in Table S1 have been calculated using the parameter estimates in S2. In order to obtain estimates for the transporter (NPF 3.1, NPF 2.14) permeabilities for the various metabolites, we used previously published data, collected from experiments with oocytes placed in an external environment of a given metabolite concentration (22, 23). We fitted these data to a mathematical model, as in (24), to calculate the corresponding membrane permeabilities. Due to difference in the structure of the transporters (23), the gibberellin metabolites can be grouped into fast (GA_{12}, GA_{15}, GA_9) and slowly (GA_4, GA_{24}) transported metabolites, and these two values appear in Tables S1-S2.

The GA_{12} delivery depends on the length of the phloem unloading zone, which has recently been imaged to be approximately the rootward half of the elongation zone (14). The constants in the Michealis-Menten term in the GA20ox-mediated biosynthesis steps been previously estimated in (15) using time course data from (25), whereas the constants related to the GA3ox-mediated step are estimated in (26).

Based on transcriptomics data (6, 27), we assume that the transcript levels of the enzymes are functions of the distance along the root, as shown in Fig. 1B of the main text. These transcriptomics data motivated us to further split the meristem into a quiescent centre zone (QCZ), which we take to be the region within 5 average meristem cell lengths of the QC ($L_{meri} = 5 \times l_{meri} = 5 \times 8.7\,\mu\text{m} = 43.5\,\mu\text{m}$). The values of the transcript levels of the enzymes, [GA20ox], [GA3ox], [GA2oxA], and [GA2oxB] are given in Supplementary Table S3.

Thus, the only unknown parameters in the model remain the synthesis/delivery rates of GA_{12} (S_{QC} and S_{phloem}) and the degradation rates for GA_4, GA_9, and GA_{12} (β_4, β_9 and β_{12} respectively). In order to obtain estimates of their values, we performed parameter surveys, varying the synthesis rate in the QCZ, the delivery rate in the phloem-unloading zone, and the three degradation rates separately and recording the square of the difference between the wild type nlsGPS1 sensor data and the model prediction:

$$f = \sum_{i=1}^{N_{data}} (\text{nlsGPS1}_i^{data} - \text{nlsGPS1}^{pred}(x_i))^2, \tag{33}$$

where N_{data} denotes the number of data points, nlsGPS1_i^{data} denotes the experimental sensor measurement i, x_i denotes the x position of data point i, and $\text{nlsGPS1}^{pred}(x_i)$ denotes the predicted sensor value at $x = x_i$.

The parameter surveys led to the parameter values presented in Table S5.

7. Quantification of the goodness-of-fit between predictions and data

To quantify the errors between predictions and data when comparing different model assumptions with different datasets, we use the Mean-Squared-Error (MSE) value:

$$\text{MSE} = \frac{1}{N_{data}} \sum_{i=1}^{N_{data}} (\text{nlsGPS1}_i^{data} - \text{nlsGPS1}^{pred}(x_i))^2, \qquad [34]$$

This measure of goodness of fit is an appropriate and standard method for comparing model predictions to noisy experimental data (see, for example (28), which discusses the root-mean-squared error (RMSE), which provides an equivalent ordering when evaluating different model assumptions). Scaling the squared error by the number of data points in each dataset enables us to make robust comparisons between the errors associated with different genetic lines (as the number of nuclei imaged varies between experimental images).

While the MSE is the standard error measure used within data-driven modelling, alternative error measures could account for the observed heteroscedasticity within the experimental data which likely arises due to the FRET data being an emission ratio. Developing a more detailed model of the sensor emission ratio that incorporates the raw fluorescent intensities from the donor and acceptor channels could be an interesting avenue for future work.

8. Effect of cytoplasmic expansion in the elongation zone

We present a simple calculation, backed up by experimental data, to motivate our assumption that the cytoplasm continues to expand in the elongation zone.

The vacuolar fraction in the meristem is approximately $\phi_{\text{meri}} = 0.35$ (8). Thus, to preserve this ratio, we must assume both the cytoplasm and the vacuole expand at the same rate, equal to the cell elongation rate in the meristem, RER_{meri}. The cytoplasmic and total cellular volumes, $V_{\text{cyt}}^{\text{meri}}$ and $V_{\text{cell}}^{\text{meri}}$, respectively, in the meristem are therefore related by

$$V_{\text{cyt}}^{\text{meri}} = (1 - \phi_{\text{meri}}) V_{\text{cell}}^{\text{meri}}. \quad [35]$$

In the elongation zone, cells increase their length (and therefore their volume) by approximately 10 times (2). Thus, we assume

$$V_{\text{cell}}^{\text{mat}} = 10 V_{\text{cell}}^{\text{meri}}, \quad [36]$$

where $V_{\text{cell}}^{\text{mat}}$ is the cell volume at the end of the elongation zone (and the start of the maturation zone). If we further assume that, in the elongation zone, cell elongation is entirely due to vacuolar expansion, then the cytoplasmic volume remains the same, i.e., using Eq. (35) and Eq. (36), the cytoplasmic volume at the end of the elongation zone, $V_{\text{cyt}}^{\text{mat}}$, is

$$V_{\text{cyt}}^{\text{mat}} = V_{\text{cyt}}^{\text{meri}} = (1 - \phi_{\text{meri}}) V_{\text{cell}}^{\text{meri}} = \frac{(1 - \phi_{\text{meri}})}{10} V_{\text{cell}}^{\text{mat}} = 0.065, \quad [37]$$

which is smaller than the value, obtained experimentally in (5, 8).

If, now, we assume that the cytoplasm continues to expand at the same rate, as the cell enters the elongation zone, then

$$\frac{1}{V_{\text{cyt}}^{\text{ez}}} \frac{\text{d}V_{\text{cyt}}^{\text{ez}}}{\text{d}t} = \text{RER}_{\text{meri}}, \quad [38]$$

where $V_{\text{cyt}}^{\text{ez}}$ is the cytoplasmic volume in the elongation zone, whereas the cell volume expands at the rate, equal to the elongation rate in the elongation zone, RER_{ez},

$$\frac{1}{V_{\text{cell}}^{\text{ez}}} \frac{\text{d}V_{\text{cell}}^{\text{ez}}}{\text{d}t} = \text{RER}_{\text{ez}}. \quad [39]$$

Dividing Eq. (38) by Eq. (39), we obtain

$$\frac{\text{d}V_{\text{cyt}}^{\text{ez}}}{\text{d}V_{\text{cell}}^{\text{ez}}} = \frac{\text{RER}_{\text{meri}}}{\text{RER}_{\text{ez}}} \frac{V_{\text{cyt}}^{\text{ez}}}{V_{\text{cell}}^{\text{ez}}}. \quad [40]$$

Integrating Eq. (40) and applying Eq. (35) and Eq. (36) gives

$$V_{\text{cyt}}^{\text{mat}} = \frac{(1 - \phi_{\text{meri}})}{10^{1 - \text{RER}_{\text{meri}}/\text{RER}_{\text{ez}}}} V_{\text{cell}}^{\text{mat}} \approx 0.1. \quad [41]$$

This value is consistent with the experimental data on the fraction of the cytoplasmic volume in the cells in the maturation zone in (5, 8).

Supplementary figures

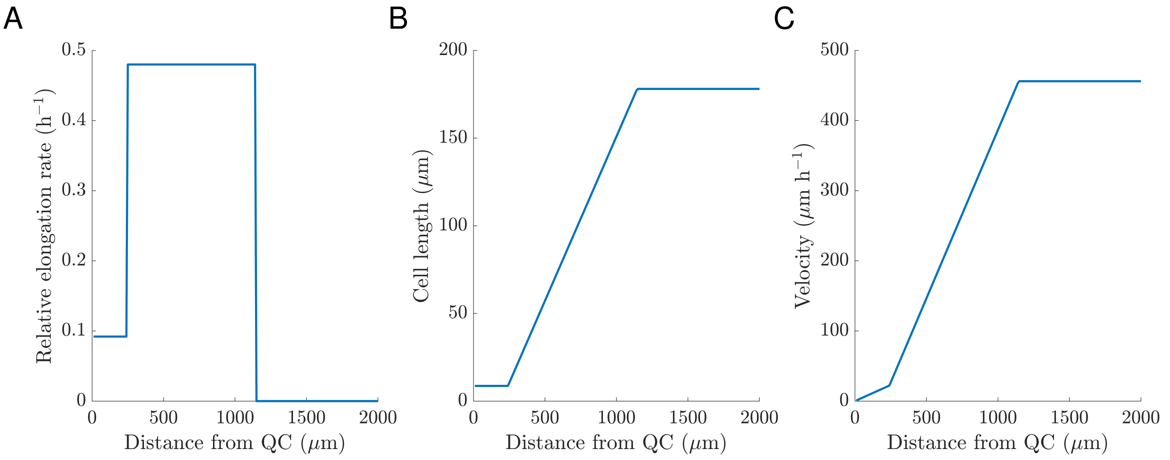

Supplementary Figure S1. Prescribed cell RER, length, and velocity. Spatial plots of the cell (**A**) relative elongation rate, (**B**) length, and (**C**) velocity.

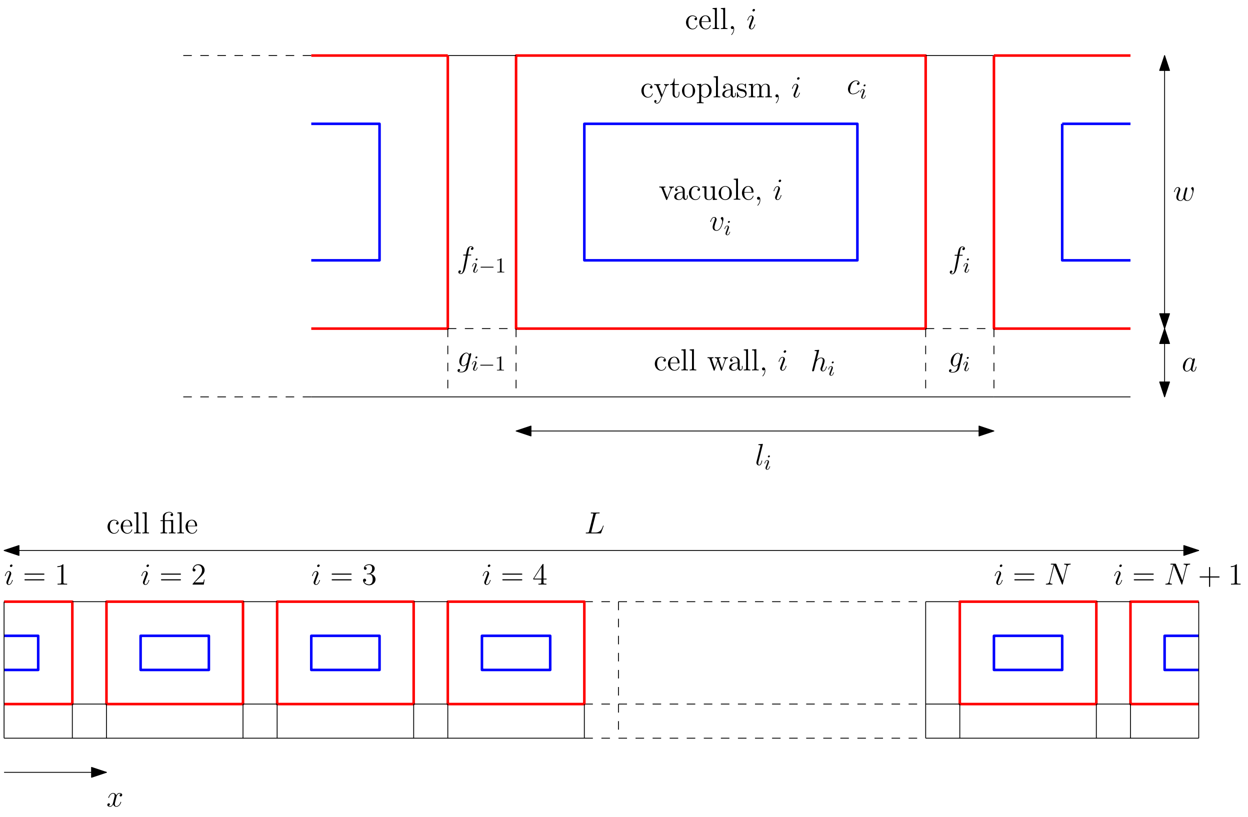

Supplementary Figure S2. Schematics of a single cell and cell file to support the explanations in Supp Info Section 1. The upper panel shows a single cell 'i' to illustrate the variable names used for the geometric parameters and concentrations in different compartments (noting that even though the apoplast is continuously connected, for analytical purposes we split it into "longitudinal" compartments aligned with the file length, "transverse" compartments between cells, and "corner" compartments, as in (1)). The lower panel shows a schematic of the cell file to show how the cell indices correspond to the continuous spatial variable x. The cytoplasmic membrane is shown in red, and the tonoplast is shown in blue.

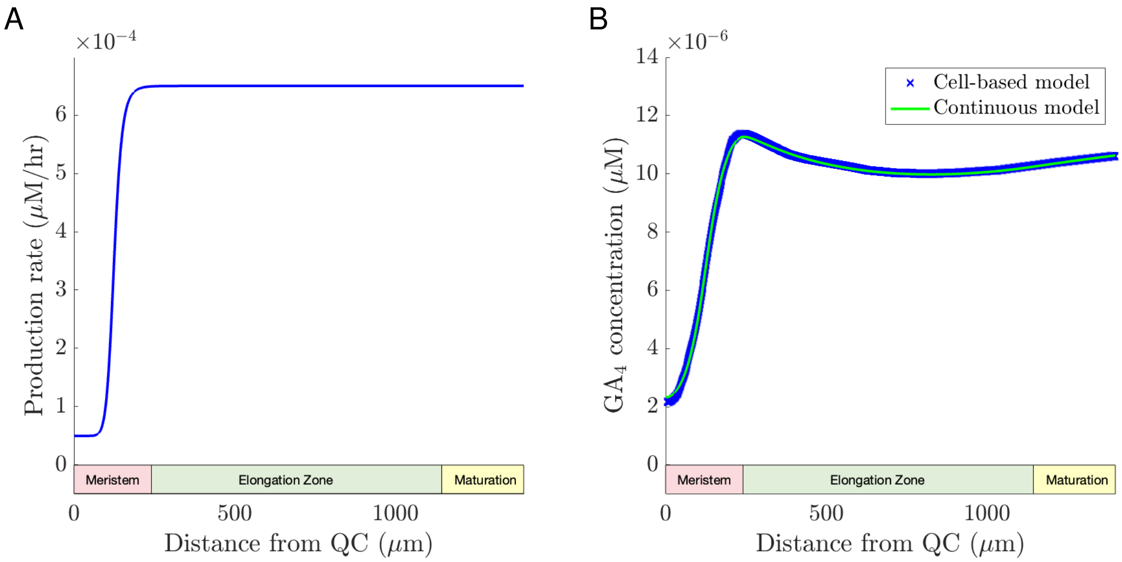

Supplementary Figure S3. Continuum model agrees well with discrete model. Comparison between the predicted GA_4 distributions for the discrete cell-based model and the continuum model, as detailed above (in SI Appendix section 3). (A) The prescribed function for the rate of GA_4 synthesis, $\sigma(x)$, given in equation (20). (B) Predicted GA_4 distribution for the continuum model (green) and the discrete model (blue).

.

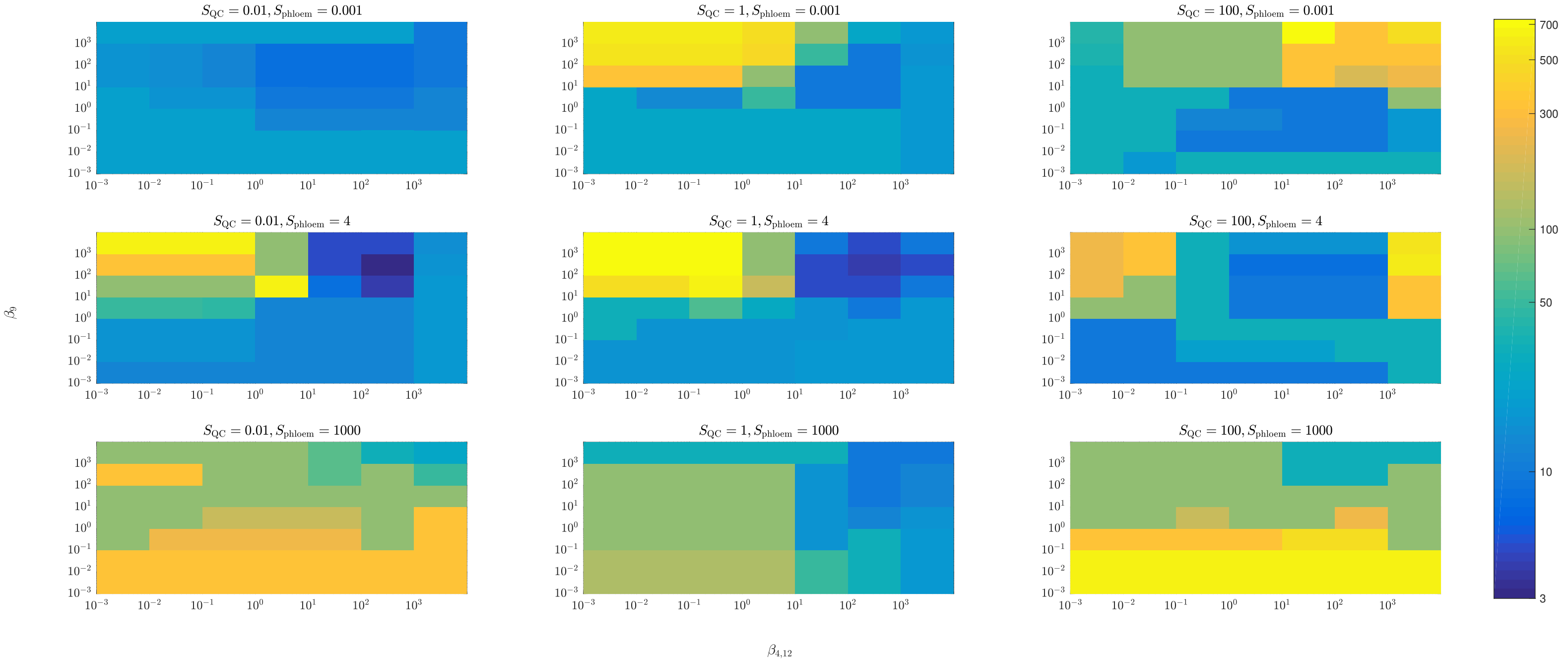

Supplementary Figure S4. Sample figures from the outputs of the parameter survey showing the effect of the unknown parameter values on the agreement between model predictions and nlsGPS1 data. The parameter survey considered 5-dimensional parameter space, surveying values of the five parameters: S_{QC}, S_{phloem}, β_{12}, β_9 and β_4. Panels show slices through this 5-dimensional parameter space, with colours showing the square of the difference between the wildtype nlsGPS1 sensor data (shown in main text, Fig 2D) and the model prediction (see equation (33)). We note these experimental data comprise 416 nlsGPS1 sensor emission ratio values all located between 0 and 500 μm from the root tip.

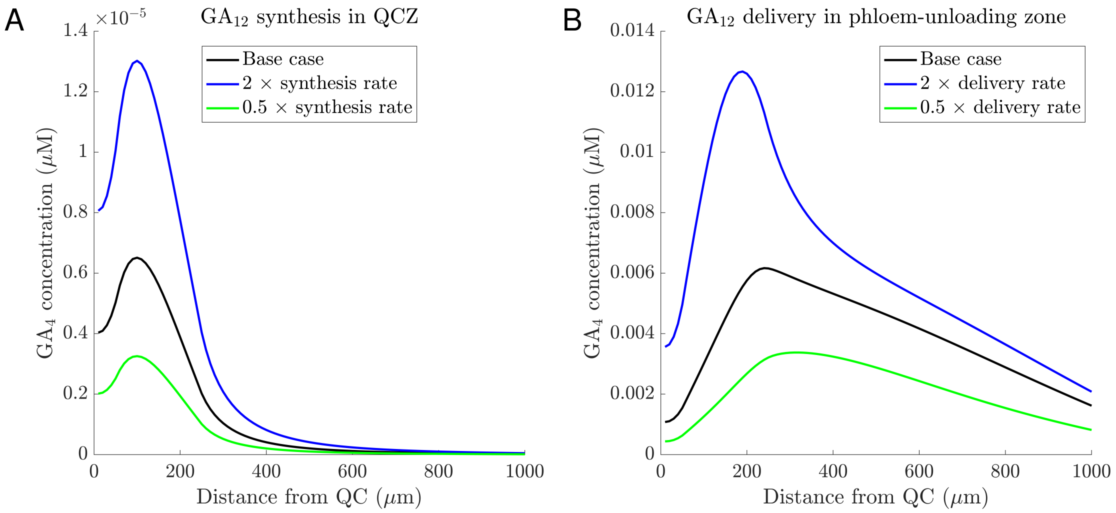

Supplementary Figure S5. Varying synthesis/delivery rates of GA_{12} scales the corresponding predicted GA_4 level. (**A**) Predicted distribution of GA_4 originating from local GA_{12} synthesis in the QCZ, with different values of the GA_{12} synthesis rate, S_{QC}. (**B**) Predicted distribution of GA_4 originating from phloem-delivered GA_{12}, with different values of the GA_{12} delivery rate, S_{phloem}.

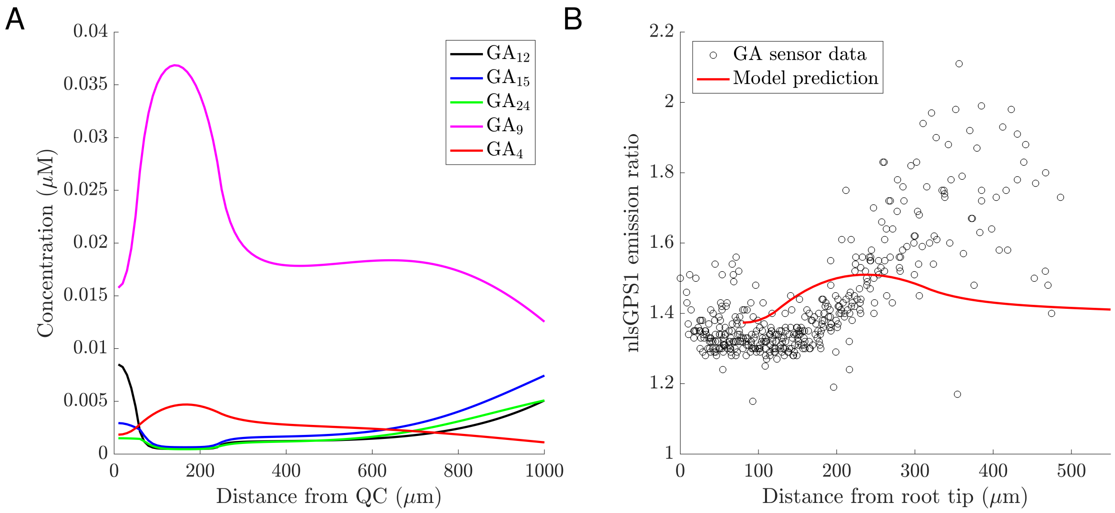

Supplementary Figure S6. With uniform delivery/synthesis of GA_{12}, the model predictions do not reproduce the GA_4 gradient observed using the nls:GPS1 sensor. (**A**) Predicted GA_{12}, GA_{15}, GA_{24}, GA_9, and GA_4 distributions with uniform GA_{12} delivery. (**B**) Comparison between model predictions and experimental data for the nlsGPS1 emission ratio. Using equation (34), we calculate the MSE$= 0.0290$ in this case.

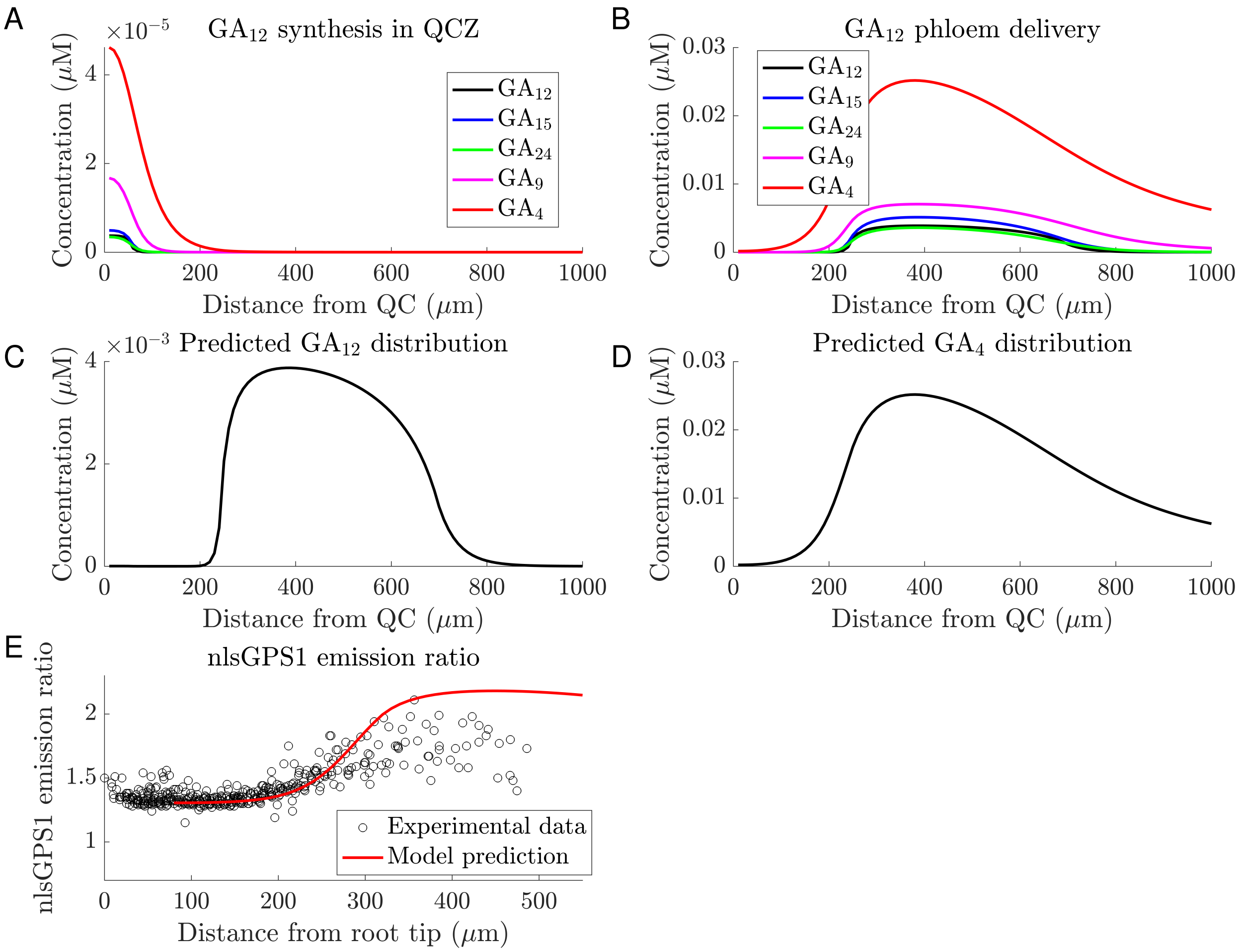

Supplementary Figure S7. With uniform enzyme transcript levels, the model predictions over-estimate the GA$_4$ levels within the elongation zone as observed using the nls:GPS1 sensor. (**A**,**B**) Predicted GA$_{12}$, GA$_{15}$, GA$_{24}$, GA$_9$, and GA$_4$ distributions with uniform enzyme levels due to (**A**) GA$_{12}$ synthesis in the QCZ and (**B**) GA$_{12}$ delivery the phloem-unloading zone. (**C**) Predicted GA$_{12}$ distribution (due to both GA$_{12}$ delivery and local synthesis). (**D**) Predicted GA$_4$ distribution (due to both GA$_{12}$ delivery and local synthesis). (**E**) Comparison between model predictions and experimental data for the nlsGPS1 emission ratio. Using equation (34), we calculate the MSE$= 0.0368$ in this case. Predicted sensor distribution is calculated from the GA$_4$ distribution shown in panel D. Parameter values are given in Tables S1 and S5, and the enzyme levels are set to unity: $[\mathrm{GA20ox}] = [\mathrm{GA3ox}] = [\mathrm{GA2oxA}] = [\mathrm{GA2oxB}] = [\mathrm{GA2oxC}] = 1$.

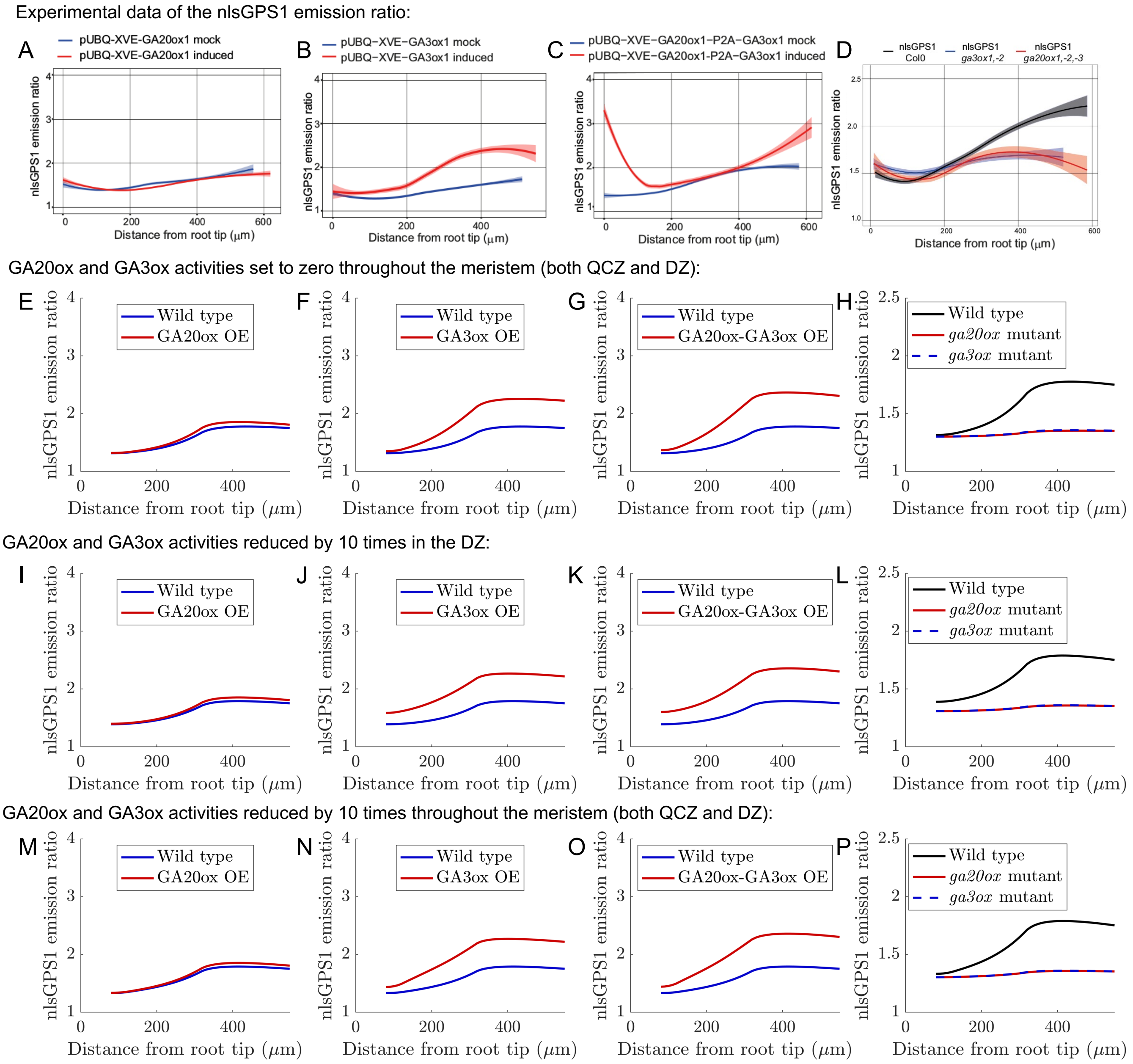

Supplementary Figure S8. nlsGPS1 distributions in different lines, with alternative model assumptions about the GA2ox and GA3ox enzyme activity. (**A-D**) Experimental nlsGPS1 data reproduced from (6) (as in Fig3A-D, reproduced here for convenience). (**E-P**) Predicted nlsGPS1 emission ratio for (**E,I,P**) GA20ox overexpression, (**F,J,N**) GA3ox overexpression, (**G,K,O**) both GA20ox and GA3ox overexpression, and (**H,L,P**) *ga20ox* and *ga3ox* loss-of-function mutants. (**E-H**) Model predictions in which the GA20ox and GA3ox activity is set to zero throughout the meristem (i.e. in both the QCZ and the DZ). (**I-L**) Model predictions in which the GA20ox and GA3ox activity is reduced by 10 times in the DZ. (**M-P**) Model predictions in which the GA20ox and GA3ox activity is reduced by 10 times throughout the meristem (i.e. in both the QCZ and the DZ). In each case, all other enzyme activities are based on the transcript level (shown in main text Fig 1B).

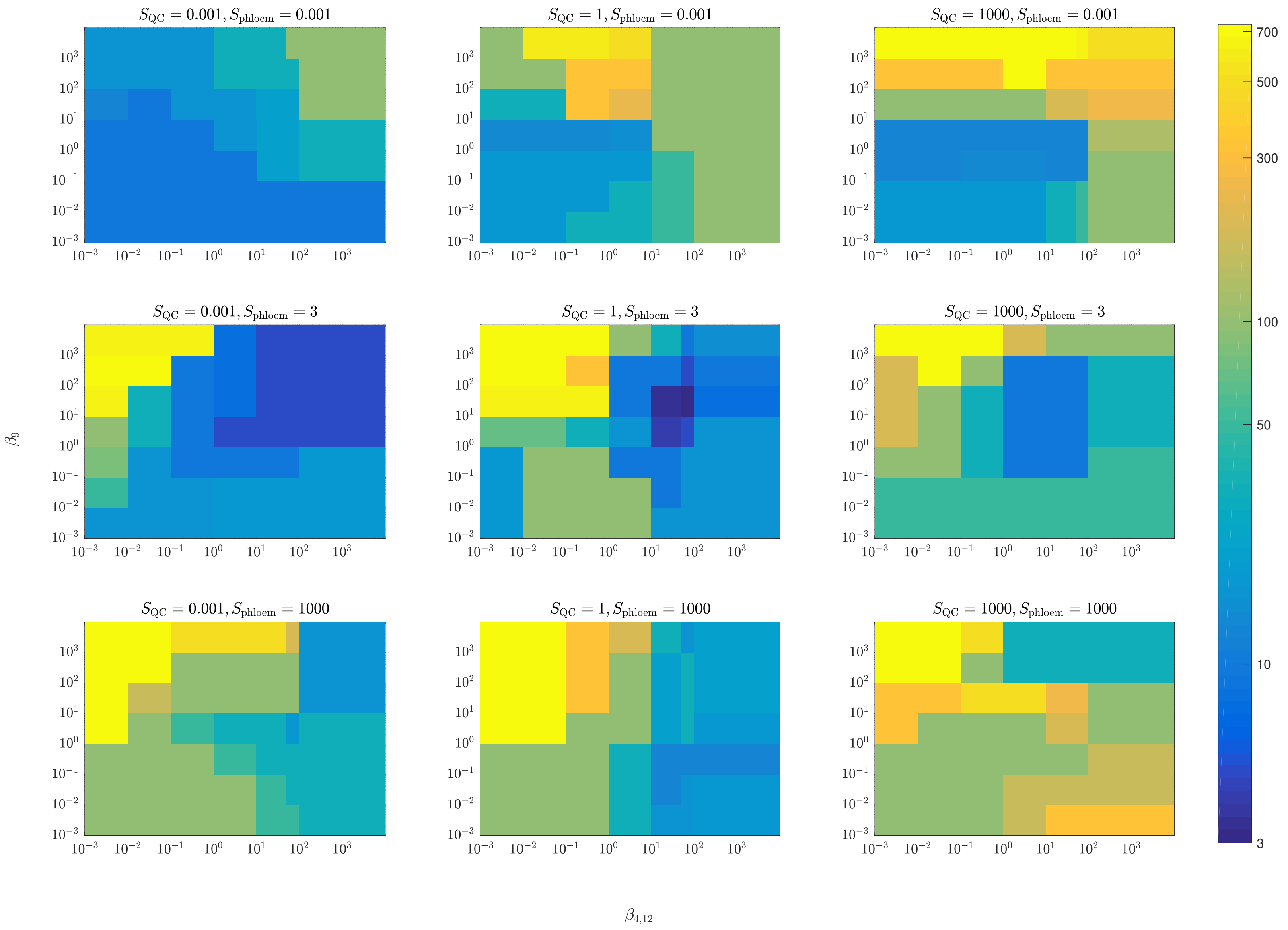

Supplementary Figure S9. Sample figures from the outputs of the parameter survey showing the effect of the unknown parameter values on the agreement between model predictions and nlsGPS1 data, assuming that GA20ox and GA3ox are inactive in the DZ. The parameter survey considered 5-dimensional parameter space, surveying values of the five parameters: S_{QC}, S_{phloem}, β_{12}, β_9 and β_4, in the range 10^{-3} and 10^3. Figures show nine slices through this 5-dimensional parameter space. Colours show the square of the difference between the wildtype nlsGPS1 sensor data (shown in main text, Fig 2D) and the model prediction for each parameter set shown (see equation (33)). We note these experimental data comprise 416 nlsGPS1 sensor emission ratio values all located between 0 and 500 μm from the root tip.

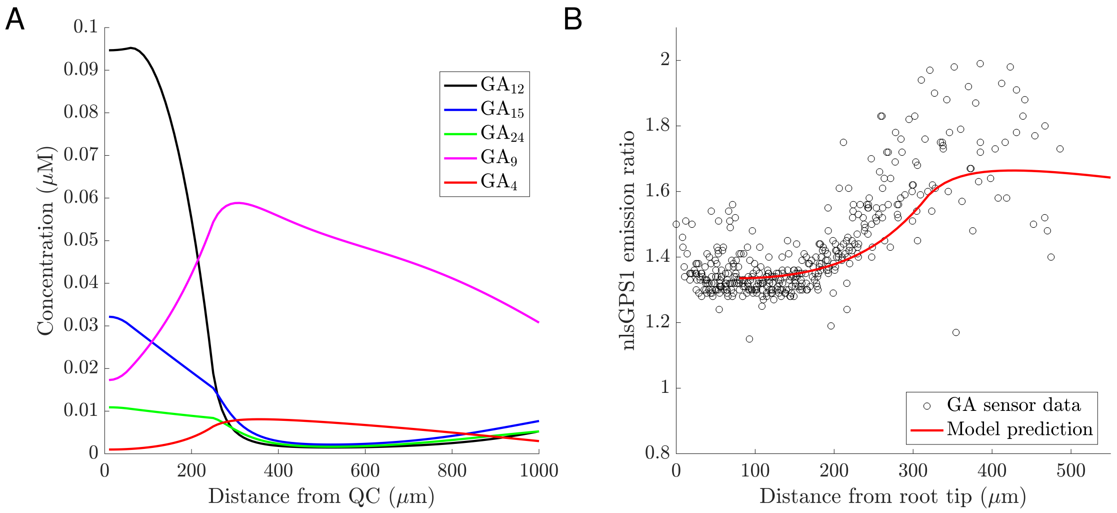

Supplementary Figure S10. With uniform delivery/synthesis of GA_{12} and GA20ox and GA3ox enzyme activities set to zero in the DZ, the model predicts a GA_4 gradient which is close to that observed via the nlsGPS1 sensor. (**A**) Predicted GA_{12}, GA_{15}, GA_{24}, GA_9, and GA_4 distributions with uniform GA_{12} synthesis/delivery. (**B**) Comparison between model predictions and experimental data for the nlsGPS1 sensor emission ratio. Using equation (34), we calculate the MSE$= 0.0133$ in this case.

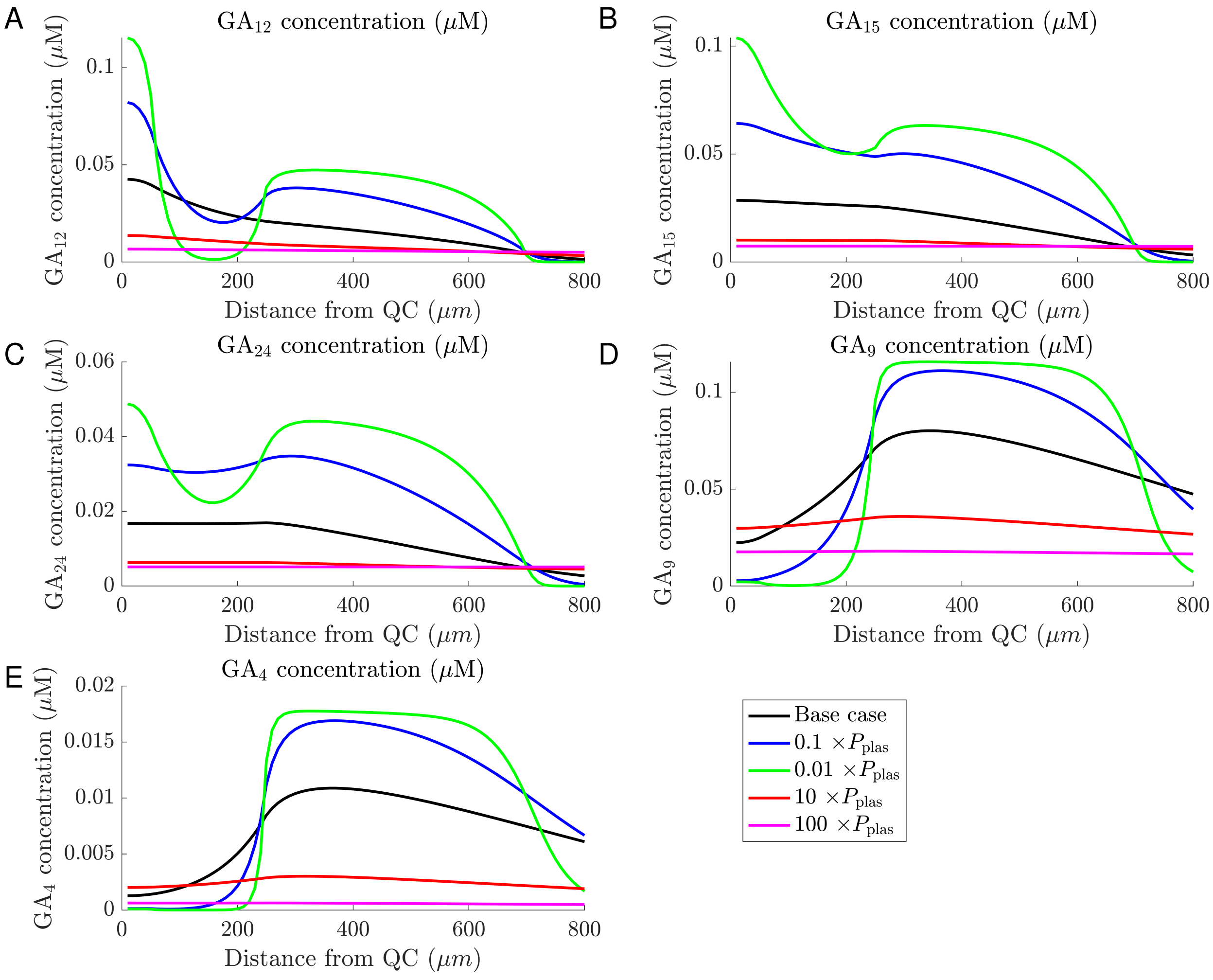

Supplementary Figure S11. Plasmodesmata have a significant effect on GA gradient. Predicted (**A**) GA_{12}, (**B**) GA_{15}, (**C**) GA_{24}, (**D**) GA_9, and (**E**) GA_4 distributions with different values for the plasmodesmatal permeability, P_{plas}.

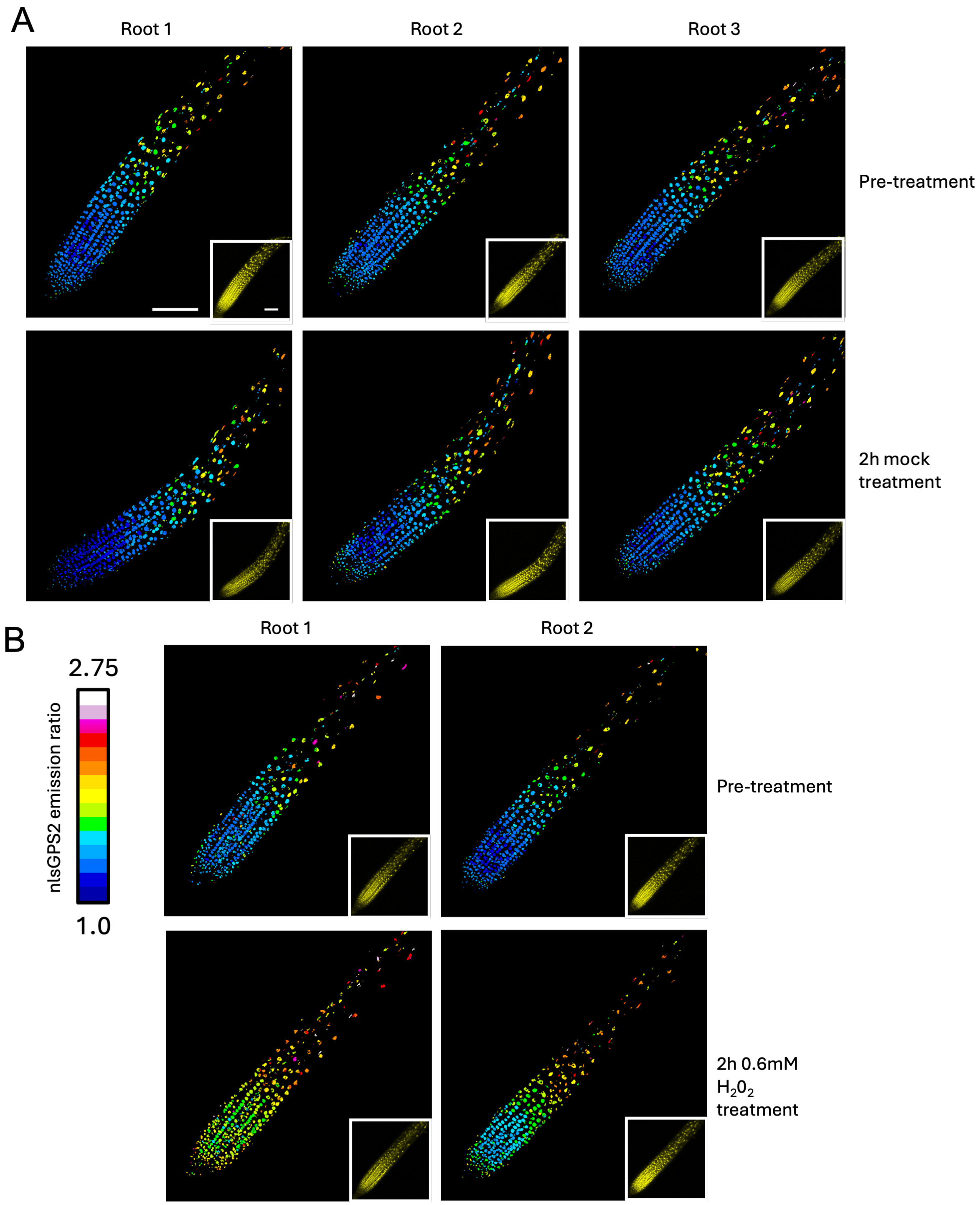

Supplementary Figure S12. Additional images from H_20_2 treatment experiments. Images of Col-0 nlsGPS2 roots. Representative images of emission ratios and YFP fluorescence (Inset) are shown of A) pre-treated and 2h mock-treated roots. B) pre-treated and 2h 0.6mM H_20_2 treated roots. Colour bar and scale bar apply to all images. Scale bar = 100μm.

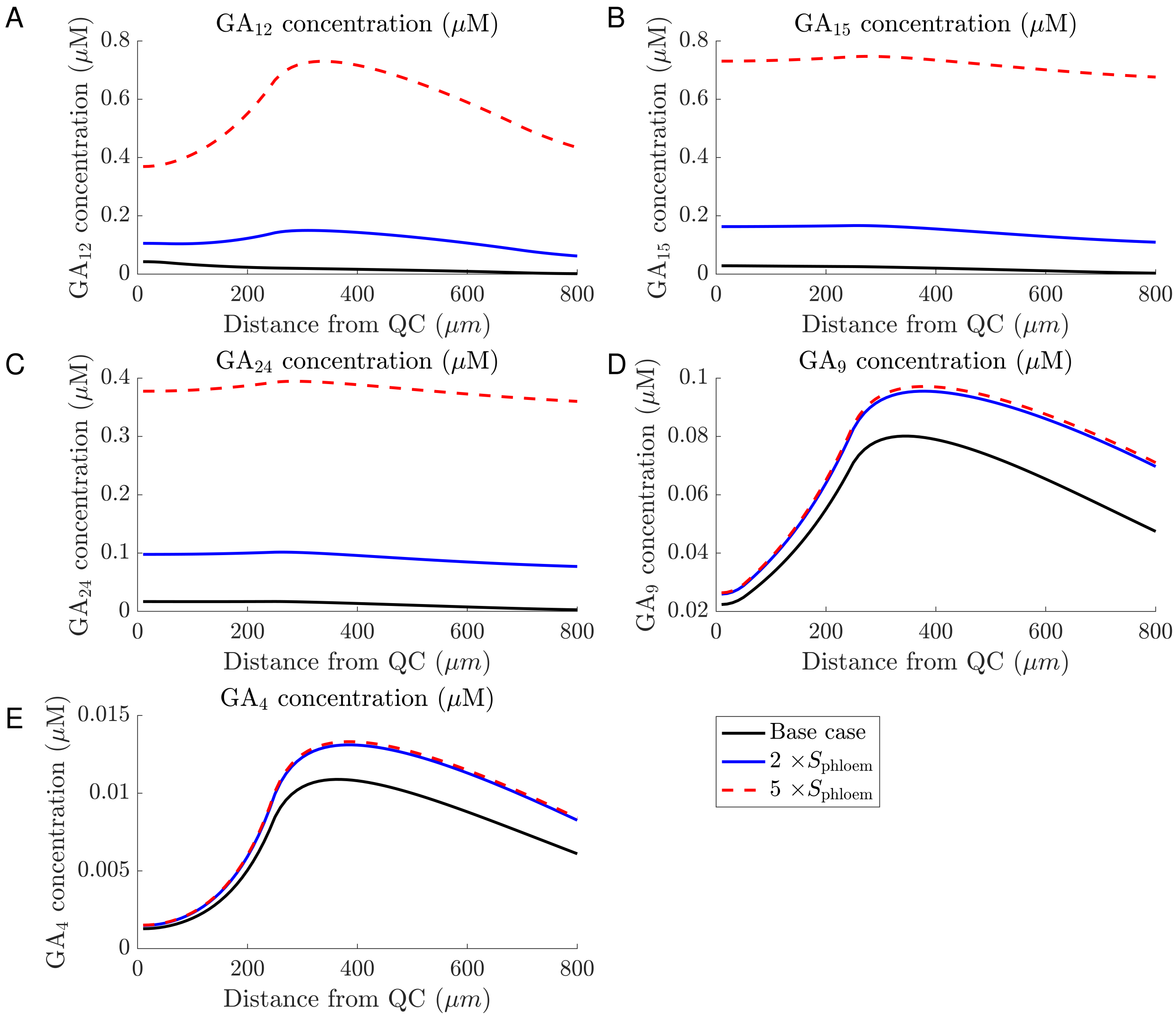

Supplementary Figure S13. Small increases in the GA_{12} delivery rate in the phloem unloading zone are predicted to increase GA_4 levels, although larger increases in delivery rate have limited effect, due to saturation of the biosynthesis steps. Predicted (**A**) GA_{12}, (**B**) GA_{15}, (**C**) GA_{24}, (**D**) GA_9, and (**E**) GA_4 distributions with different values for the GA_{12} delivery rate, S_{phloem}.

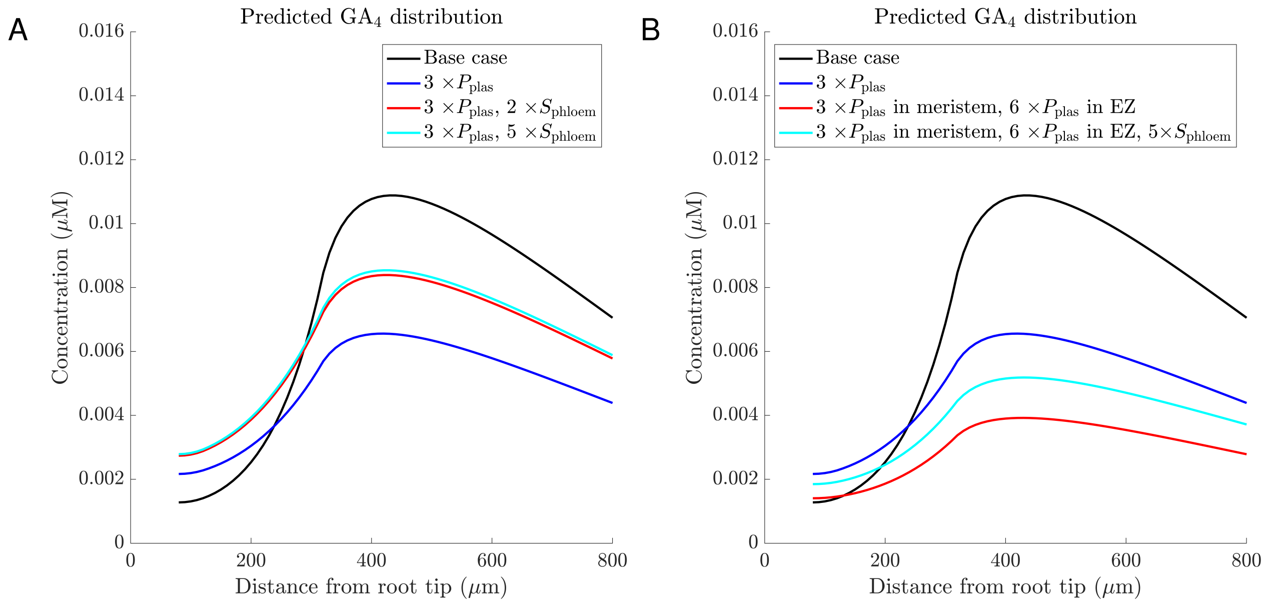

Supplementary Figure S14. Predicted GA_4 distributions due to hypothesised changes that may be caused by the H_2O_2 treatment. (**A**) Predicted GA_4 distributions with both increased plasmodesmatal permeability, P_{plas}, and increased GA_{12} phloem delivery, S_{phloem}. (**B**) Predicted GA_4 distributions with higher P_{plas} in the EZ and maturation zones (hypothesised to be due to increased permeability to external treatments) combined with increased GA_{12} delivery.

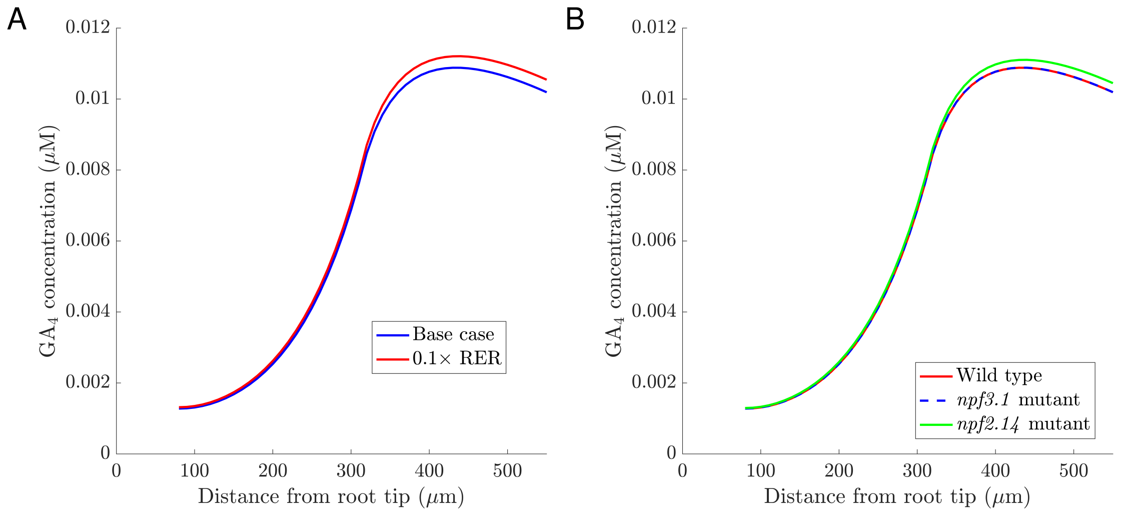

Supplementary Figure S15. Dilution and NPF-mediated transport have limited effect on the predicted GA_4 distribution. (**A**) Predicted GA_4 distribution with the relative elongation rates reduced by ten times (i.e. $\mathrm{RER}_{\mathrm{meri}} = 0.0092$ and $\mathrm{RER}_{\mathrm{EZ}} = 0.048$). (**B**) Predicted GA_4 distribution in wildtype, the *npf3.1* mutant ($P_{\mathrm{imp}} = 0$) and the *npf2.14* mutant ($P_{\mathrm{exp}} = 0$). In each case, the remaining parameter values are given in Tables S1, S3 and S5.

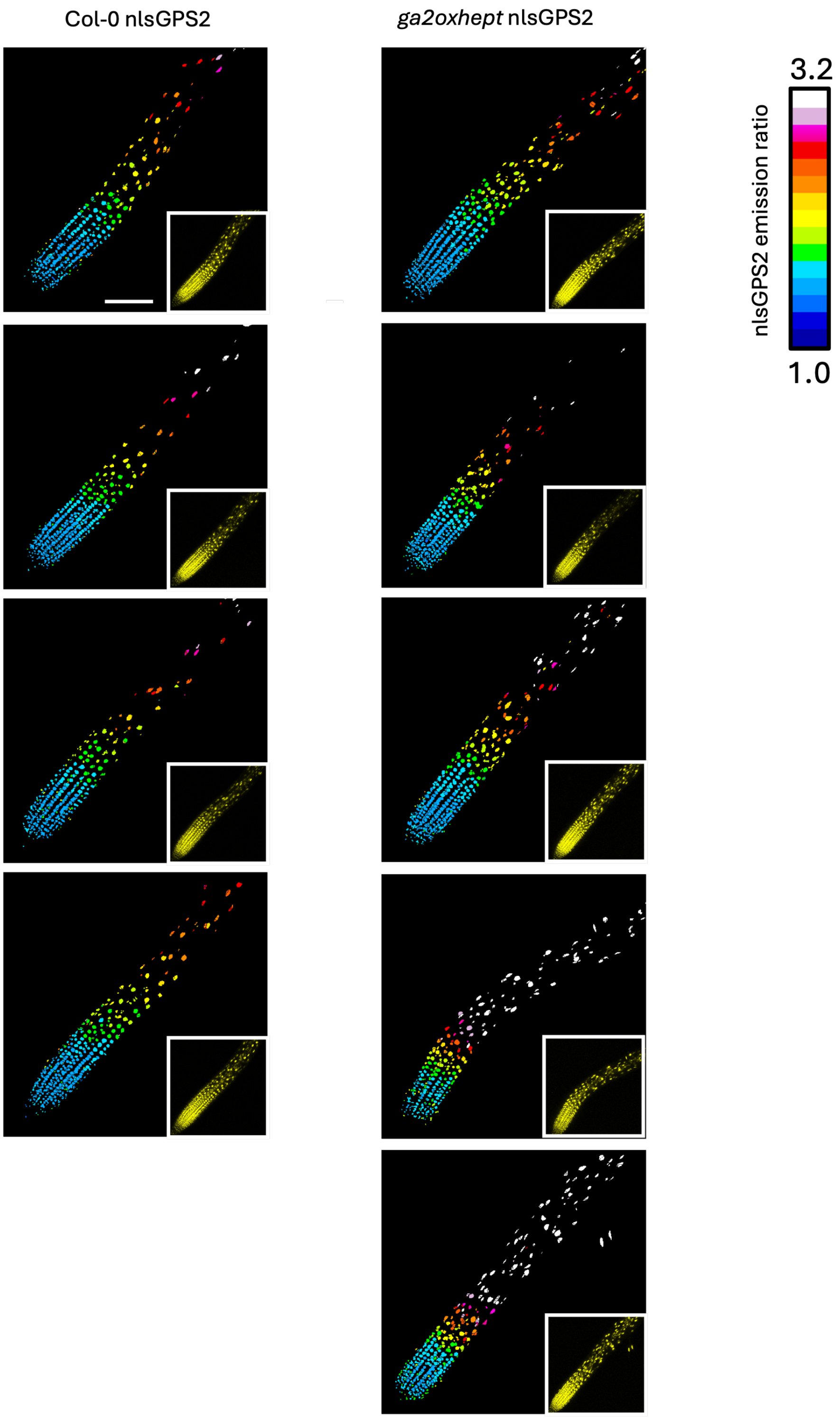

Supplementary Figure S16. Additional images of Col-0 nlsGPS2 and ga2oxhept nlsGPS2 roots. Representative images of emission ratios and YFP fluorescence (Inset) are shown. Colour bar and scale bar apply to all images. Scale bar = 100μm.

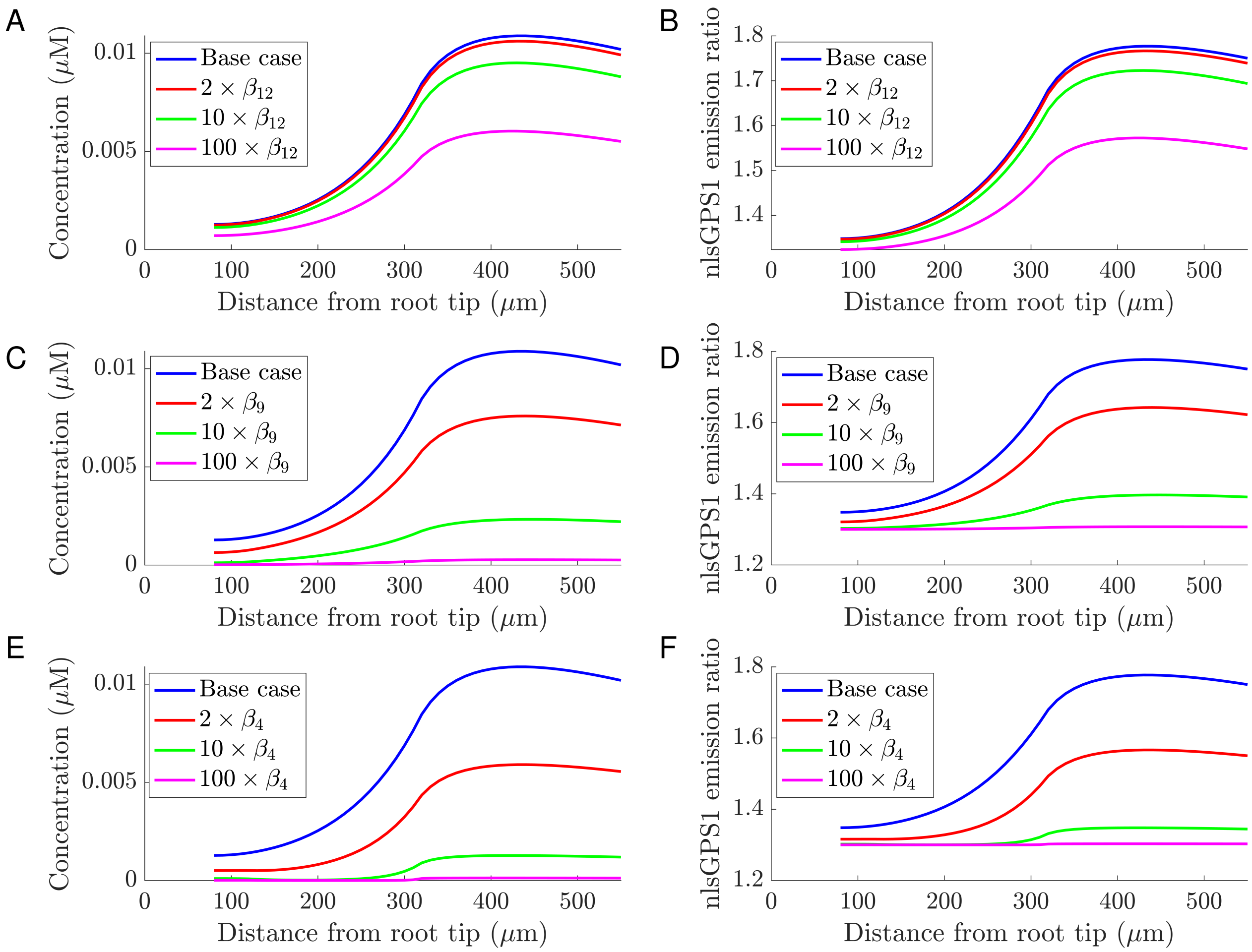

Supplementary Figure S17. Effect of increasing the GA2ox-mediated degradation rates on the predicted GA_4 and nlsGPS1 distributions. (**A, B**) Model predictions increasing the GA_{12} degradation rate, β_{12}. (**C,D**) Model predictions increasing the GA_9 degradation rate, β_9. (**E,F**) Model predictions increasing the GA_4 degradation rate, β_4. We note that increasing a degradation rate to $10\times$ or $100\times$ would represent the corresponding GA2ox overexpression line.

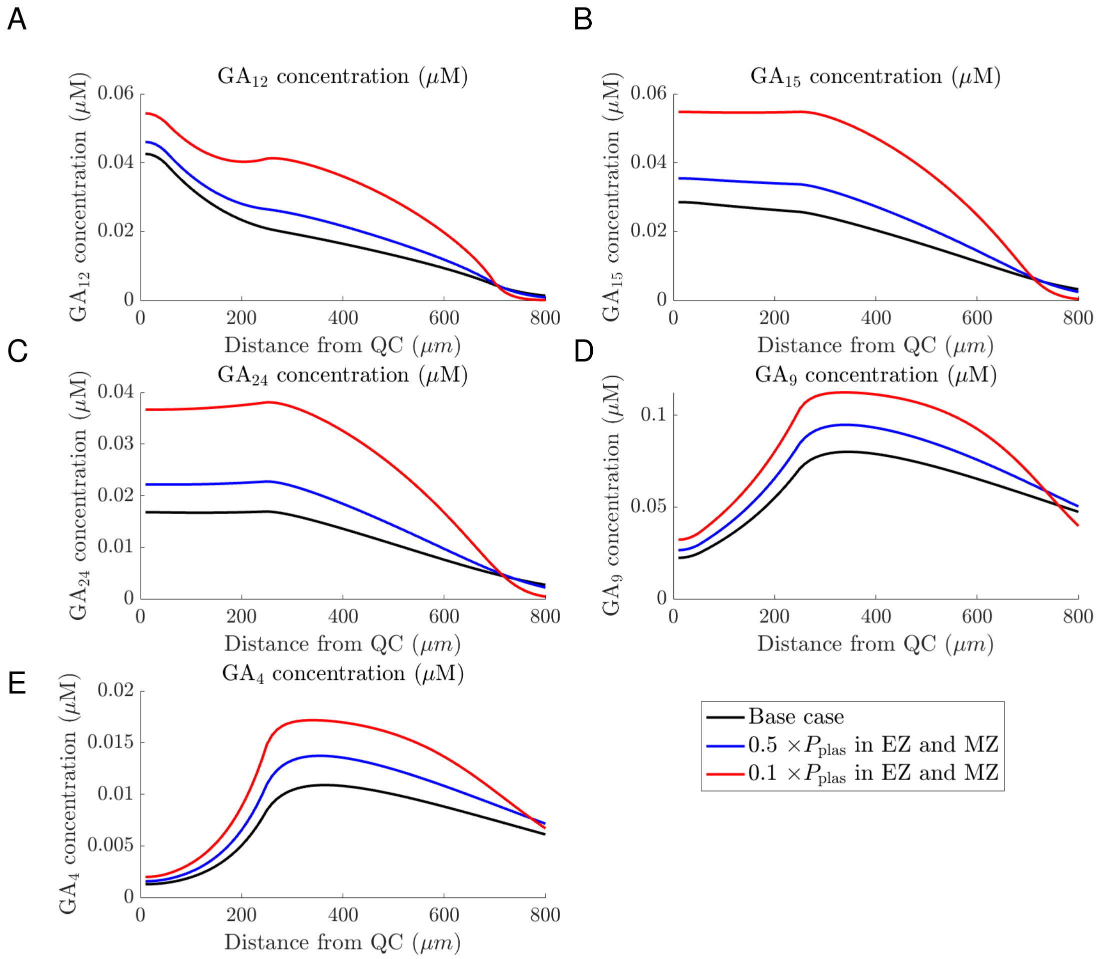

Supplementary Figure S18. A lower plasmodesmatal permeability in the elongation and maturation zones is predicted to increase the GA_4 gradient. Simulations with spatial variations in the plasmodesmatal permeability (which characterises the plasmodesmatal diffusion between adjacent cells within the cell file). We present simulations with reduced plasmodesmatal permeability in the elongation and maturation zones, to represent hypothesised reductions in plasmodesmatal diffusion due to callose deposition (29, 30)) or increased cell-wall thickness (20). Predictions of the distributions of (**A**) GA_{12}, (**B**) GA_{15}, (**C**) GA_{24}, (**D**) GA_9, and (**E**) GA_4 with different values for the plasmodesmata permeability in the elongation and maturation zones.

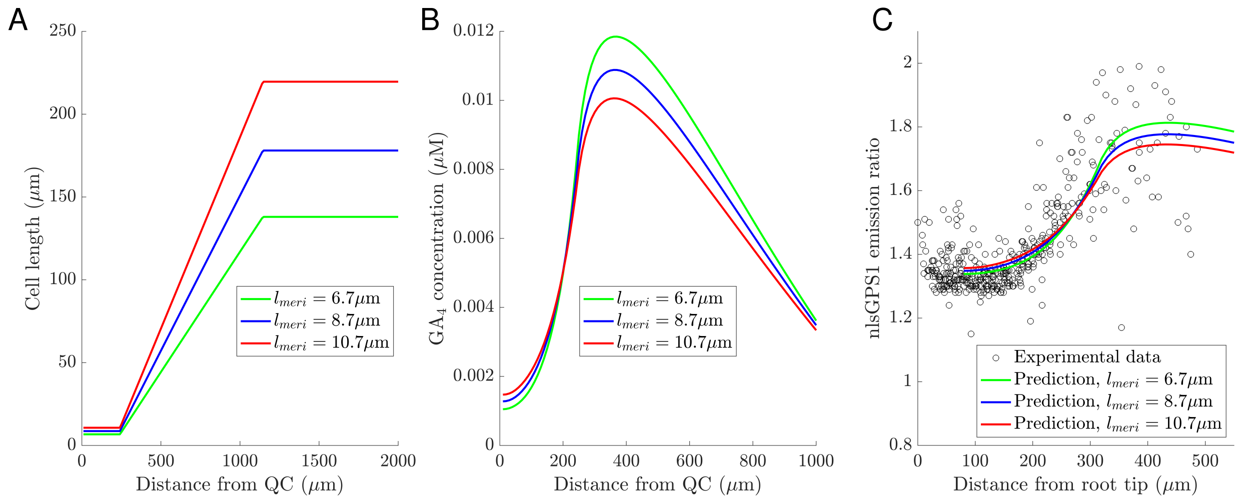

Supplementary Figure S19. Increased cell lengths lead to a small reduction in the predicted GA_4 gradient. (A) Resulting distribution of cell lengths across the growth zone for different average meristem cell lengths. (B) Predicted GA_4 distributions with different values for the prescribed average meristem cell length, l_{meri}. (B) Predicted distribution of the nlsGPS1 emission ratios with different values for the prescribed average meristem cell length, l_{meri}, together with nlsGPS1 experimental data (as shown in Fig 2D).

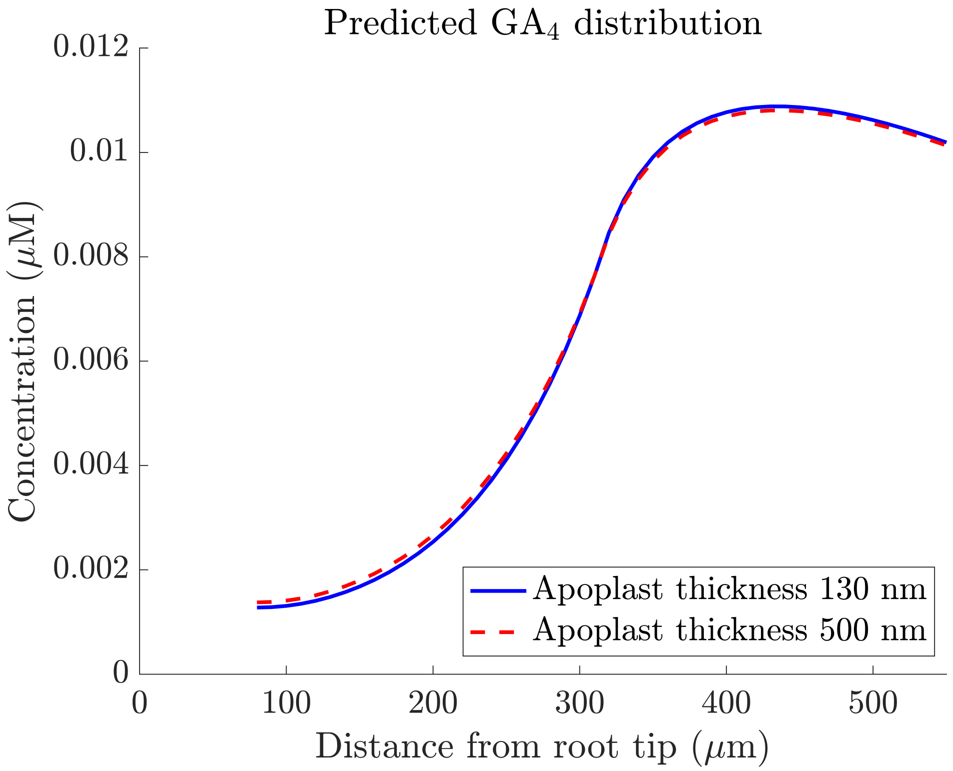

Supplementary Figure S20. Apoplast thickness has little effect on the predicted GA_4 distribution.

359 **Supplementary tables**

Parameter	Description	Value	Reference
l_0	initial cell length in meristem	$6\,\mu$m	(2)
$L_{\rm file}$	file length	$1300\,\mu$m	(6)
$L_{\rm QC}$	length of quiescent centre zone	$43.5\,\mu$m	(31)
$L_{\rm phloem}$	length of phloem-unloading zone	$450\,\mu$m	(14)
$L_{\rm meri}$	length of meristem	$240\,\mu$m	(6)
$L_{\rm ez}$	length of elongation zone	$900\,\mu$m	(6)
$\phi_{\rm meri}$	vacuolar fraction in meristem	0.35	(8)
$\phi_{\rm mat}$	vacuolar fraction in maturation zone	0.9	(8)
$\mathrm{RER}_{\rm meri}$	cell elongation rate in meristem	$0.092\,\mathrm{h}^{-1}$	(3)
$\mathrm{RER}_{\rm ez}$	cell elongation rate in elongation zone	$0.48\,\mathrm{h}^{-1}$	(3)
w	cell width	$10\,\mu$m	(32)
a	apoplast thickness	$0.13\,\mu$m	(18)
$D_{\rm apo}$	apoplastic diffusivity	$32\,\mu\mathrm{m}^2\mathrm{s}^{-1}$	(17)
$P_{\rm plas}$	plasmodesmatal permeability	$3.3\,\mu\mathrm{m\,s}^{-1}$	(19)
λ_{12}	rate of conversion from GA_{12} to GA_{15}	$2580\,\mu\mathrm{M}^{-1}\mathrm{h}^{-1}$	(15)
λ_{15}	rate of conversion from GA_{15} to GA_{24}	$1920\,\mu\mathrm{M}^{-1}\mathrm{h}^{-1}$	(15)
λ_{24}	rate of conversion from GA_{24} to GA_9	$2700\,\mu\mathrm{M}^{-1}\mathrm{h}^{-1}$	(15)
λ_9	rate of conversion from GA_9 to GA_4	$408\,\mu\mathrm{M}^{-1}\mathrm{h}^{-1}$	(26)
κ_{12}	constant in Hill function in Eq. (28a)	$0\,\mu\mathrm{M}^{-1}\mathrm{h}^{-1}$	(15)
κ_{15}	constant in Hill function in Eq. (28a)	$0\,\mu\mathrm{M}^{-1}\mathrm{h}^{-1}$	(15)
κ_{24}	constant in Hill function in Eq. (28a)	$500\,\mu\mathrm{M}^{-1}\mathrm{h}^{-1}$	(15)
κ_9	constant in Hill function in Eq. (28e)	$1\,\mu\mathrm{M}^{-1}\mathrm{h}^{-1}$	(26)
$P_{\rm ca}$	$B_1P_{\rm pass}+B_2P_{\rm imp}$	$0.0064/0.0054\,\mu\mathrm{m\,s}^{-1}$	[$\star$]
$P_{\rm ac}$	$A_1P_{\rm pass}+A_2P_{\rm imp}$	$0.629/0.714\,\mu\mathrm{m\,s}^{-1}$	[$\star$]
$P_{\rm cv}$	$B_1P_{\rm pass}+B_3P_{\rm exp}$	$0.940/0.0493\,\mu\mathrm{m\,s}^{-1}$	[$\star$]
$P_{\rm vc}$	$C_1P_{\rm pass}+C_3P_{\rm exp}$	$0.295/0.159\,\mu\mathrm{m\,s}^{-1}$	[$\star$]

Supplementary Table S1. Physical model parameter estimates obtained from the cited literature. The quantities with stars [$\star$] are calculated from the quantities and formulae listed in the Table S2. Left-hand-side estimates pertain to GA_4 and GA_{24}, whereas the corresponding estimates on the right-hand side pertain to GA_{12}, GA_{15}, and GA_9.

Parameter	Description	Value	Reference
$\mathrm{pH}_{\mathrm{cyt}}$	cytoplasmic pH	7.6	(33)
$\mathrm{pH}_{\mathrm{apo}}$	apoplastic pH	5.3	(34)
$\mathrm{pH}_{\mathrm{vac}}$	vacuolar pH	5.8	(35)
pK	equilibrium dissociation constant	4.2/4.3	(36)
V_{mem}	potential across cell membrane	$-120\,\mathrm{mV}$	(33, 34)
V_{ton}	potential across tonoplast	$-30\,\mathrm{mV}$	(33, 37)
T	absolute temperature	$300\,\mathrm{K}$	(34)
P_{pass}	passive permeability	$0.333/4.72\,\mu\mathrm{m\,s^{-1}}$	(22, 24)
P_{imp}	importer permeability	$0.139/0.0667\,\mu\mathrm{m\,s^{-1}}$	(22, 24)
P_{exp}	exporter permeability	$0.556/0.0278\,\mu\mathrm{m\,s^{-1}}$	(23, 24)
F_D	Faraday's constant	$96500\,\mathrm{C\,mol^{-1}}$	(34)
R	universal gas constant	$8.31\,\mathrm{J\,mol^{-1}K^{-1}}$	(34)
A_1	$1/(1+10^{\mathrm{pH_{apo}}-\mathrm{pK}})$	0.0736/0.0909	[$\star$]
A_2	$(F_D V_{\mathrm{mem}}/RT)(1-A_1)/(\mathrm{e}^{F_D V_{\mathrm{mem}}/RT}-1)$	4.34/4.26	[$\star$]
B_1	$1/(1+10^{\mathrm{pH_{cyt}}-\mathrm{pK}})$	0.000501/0.000398	[$\star$]
B_2	$-(F_D V_{\mathrm{mem}}/RT)(1-B_1)/(\mathrm{e}^{-F_D V_{\mathrm{mem}}/RT}-1)$	0.0451/0.045	[$\star$]
B_3	$(F_D V_{\mathrm{ton}}/RT)(1-B_1)/(\mathrm{e}^{F_D V_{\mathrm{ton}}/RT}-1)$	1.69/1.69	[$\star$]
C_1	$1/(1+10^{\mathrm{pH_{vac}}-\mathrm{pK}})$	0.0245/0.0307	[$\star$]
C_3	$-(F_D V_{\mathrm{ton}}/RT)(1-C_1)/(\mathrm{e}^{-F_D V_{\mathrm{ton}}/RT}-1)$	0.516/0.513	[$\star$]

Supplementary Table S2. Physical parameter estimates used in calculating the model parameter values related to transport (listed and marked with stars in Table S1). The quantities with stars here [$\star$] are calculated from other quantities in this table according to the corresponding formula. The permeability estimates are obtained from oocyte experiments, as in our previous work (24). Their values, the pK values, and the related constants on the left-hand side pertain to GA_4 and GA_{24}, whereas the corresponding estimates on the right-hand side pertain to GA_{12}, GA_{15}, and GA_9. Here, for concreteness, we have taken P_{imp} to refer to the permeability estimated from oocyte experiments with NPF3.1. P_{exp} refers to the permeability estimated from data for NPF2.14.

Region	[GA20ox]	[GA3ox]	[GA2oxA]	[GA2oxB, C]
quiescent-centre zone (QCZ)	0.01	0.01	0.08	1
division zone (DZ)	1	0.01	0.04	0.2
elongation zone (EZ)	0.5	0.025	0.08	0.65
maturation zone (MZ)	0.05	0.0025	0.08	10

Supplementary Table S3. Enzyme transcript levels assumed in the model, based on transcriptomics data in (6, 27). [GA20ox] and [GA3ox] in the DZ are either set to zero or reduced to $0.1\times$ in the simulations with zero or reduced biosynthesis enzyme activity in the DZ respectively.

Cell length, l	Time scale for intracellular diffusion, l^2/D_c		Time scale for plasmodesmatal diffusion, l/P_{plas}
	$D_c = 176\,\mu$m^2/s	$D_c = 570\,\mu$m^2/s	
$8.7\,\mu$m	0.43 s	0.14 s	2.6 s
$40\,\mu$m	9.09 s	2.96 s	12.12 s
$180\,\mu$m	184.09 s	60s	54 s

Supplementary Table S4. Calculations of approximate time scales for intracellular diffusion within the cytoplasm and plasmodesmatal diffusion, considering meristematic cells, with a cell length of $l = 8.7\,\mu\mathrm{m}$, cells within the early elongation zone, with a cell length of $l = 40\,\mu\mathrm{m}$, and mature cells, with cell length of $l = 180\,\mu\mathrm{m}$. The plasmodesmatal permeability is take to be $P_{\mathrm{plas}} = 3.3\mu\mathrm{m/s}$ (19). The cytoplasmic diffusivity, D_c has not been well-characterised. For the hormone auxin, previous studies have taken the cytoplasmic diffusivity to be the aqueous value, $D_c \approx 670\,\mu\mathrm{m}^2/$s (38) whereas others use a smaller value, $D_c \approx 220\,\mu\mathrm{m}^2/$s (19). Noting that auxin is a smaller molecule that GA_4 (with molecular mass of 175 Daltons and 332 Daltons respectively), the Stokes-Einstein relationship suggests that the diffusivity of GA_4 would be approximately 80% of that of auxin. Given these considerations, we therefore give values for the time scale for intracellular diffusion with two possible values of D_c. For the cells within the meristem, the time scale for intracellular diffusion is smaller than that for plasmodesmatal diffusion, suggesting that the intracellular diffusion is sufficient to smooth out intracellular gradients in this zone. For elongated cells, the difference between the calculated time scales of intracellular diffusion and plasmodesmatal diffusion depends on the value of the cytoplasmic diffusion rate, D_c. Should auxin diffuse in the cytoplasm as it does in water, $D_c = 570\,\mu$m^2/s, then intracellular diffusion occurs faster than plasmodesmatal diffusion for cells within the early elongation zone, but on a similar time scale as plasmodesmatal diffusion for mature cells. However, if the composition of the cytoplasm leads to a slower cytoplasmic diffusion, $D_c = 176\,\mu$m^2/s, then the time scale for intracellular diffusion would be similar to that of plasmodesmatal diffusion for cells within the early elongation zone, but would be larger than that of plasmodesmatal diffusion for the mature cells. These values suggest that intracellular gradients may be present within the elongated cells. More detailed data on cytoplasmic diffusion rates would help clarify the appropriate model assumption and role of intracellular diffusivity.

Parameter	Description	Value
S_{QC}	production rate of GA_{12} in QCZ	$0.01/1\,\mu\mathrm{M\,h}^{-1}$
S_{phloem}	delivery rate of GA_{12} in phloem-unloading zone	$4/3\,\mu\mathrm{M\,h}^{-1}$
β_{12}	rate of degradation of GA_{12}	$100/90\,\mathrm{h}^{-1}$
β_9	rate of degradation of GA_9	$80/20\,\mathrm{h}^{-1}$
β_4	rate of degradation of GA_4	$100/90\,\mathrm{h}^{-1}$

Supplementary Table S5. Parameter estimates obtained from the parameter survey. The left-hand values are for the initial simulations where enzyme activities depend solely on the transcript level, and the right-hand values are for the simulations where we assumed no activity of GA20ox and GA3ox in the DZ.

Case	Wild type	GA20ox OE	GA3ox OE	GA20oxGA3ox OE	ga20ox KO	ga3ox KO	All datasets
Number of data points	304	301	217	353	192	118	1485
Original	0.0154	0.0119	0.1285	0.1018	0.0543	0.0811	0.0620
No activity in DZ	0.0105	0.0262	0.0816	0.0972	0.0667	0.0778	0.0573
No activity in QCZ and DZ	0.0103	0.0264	0.0830	0.1063	0.0672	0.0786	0.0598
Reduced activity in DZ	0.0124	0.0282	0.1011	0.1019	0.0647	0.0758	0.0616
Reduced activity in QCZ and DZ	0.0101	0.0278	0.0872	0.1052	0.0656	0.0770	0.0601

Supplementary Table S6. Quantification of the comparison between model predictions and nlsGPS1 data for the results shown in Figures 2-4 (main text) and Figure S8 above. For each of the five cases and each of the six genetic lines, we calculated the distance between the model prediction and data using the mean-squared-error (MSE) given in equation (34). The final column gives the mean-squared-error between predictions and data over all the data points in all six datasets. KO = knockout/loss-of function mutant.

References

1. K. B. Kiradjiev and L. R. Band. Multiscale asymptotic analysis reveals how cell growth and subcellular compartments affect tissue-scale hormone transport. *Bull. Math. Biol.*, 85(10):101, 2023. .
2. T. Goh et al. In-depth quantification of cell division and elongation dynamics at the tip of growing *Arabidopsis* roots using 4D microscopy, AI-assisted image processing and data sonification. *Plant Cell Physiol.*, page pcad105, 2023. .
3. C. M. van der Weele et al. A new algorithm for computational image analysis of deformable motion at high spatial and temporal resolution applied to root growth. roughly uniform elongation in the meristem and also, after an abrupt acceleration, in the elongation zone. *Plant Physiol.*, 132(3):1138–1148, 2003. .
4. P. B. Green. Growth and cell pattern formation on an axis: Critique of concepts, terminology, and modes of study. *Bot. Gaz.*, 137(3):187–202, 1976. .
5. K. Dünser et al. Extracellular matrix sensing by FERONIA and Leucine-Rich Repeat Extensins controls vacuolar expansion during cellular elongation in *Arabidopsis thaliana*. *EMBO J.*, 38(7):e100353, 2019. .
6. A. Rizza et al. Differential biosynthesis and cellular permeability explain longitudinal gibberellin gradients in growing roots. *Proc. Natl. Acad. Sci.*, 118(8):e1921960118, 2021. .
7. L. R. Band et al. Growth-induced hormone dilution can explain the dynamics of plant root cell elongation. *Proc. Natl. Acad. Sci.*, 109(19):7577–7582, 2012. .
8. K. Dünser et al. Endocytic trafficking promotes vacuolar enlargements for fast cell expansion rates in plants. *eLife*, 11:e75945, 2022. .
9. P. L. Webster and R. D. MacLeod. Characteristics of root apical meristem cell population kinetics: A review of analyses and concepts . *Environ. Exp. Bot.*, 120(4):335–358, 1980. .
10. V. B. Ivanov. *Root growth responses to chemicals*. Harwood Academic Publishers, 1994.
11. G. T. S. Beemster and T. I. Baskin. Analysis of cell division and elongation underlying the developmental acceleration of root growth in *Arabidopsis thaliana*. *Plant Physiol.*, 116(4):1515–1526, 1998. .
12. T. I. Baskin et al. Positioning the root elongation zone is saltatory and receives input from the shoot. *iScience*, 23(7):101309, 2020. .
13. I. Rieu, S. Eriksson, S. J. Powers, F. Gong, J. Griffiths, L. Woolley, R. Benlloch, O. Nilsson, S. G. Thomas, P. Hedden, and A. L. Phillips. Genetic analysis reveals that C19-GA 2-oxidation is a major gibberellin inactivation pathway in *Arabidopsis*. *The Plant Cell*, 20(9):2420–2436, 09 2008. .
14. T. J. Ross-Elliott et al. Phloem unloading in *Arabidopsis* roots is convective and regulated by the phloem-pole pericycle. *eLife*, 6:e24125, 2017. .
15. L. R. Band and S. P. Preston. Parameter inference to motivate asymptotic model reduction: An analysis of the gibberellin biosynthesis pathway. *J. Theor. Biol.*, 457:66–78, 2018. .
16. A.M. Middleton et al. Mathematical modeling elucidates the role of transcriptional feedback in gibberellin signaling. *Proc Natl Acad Sci USA*, 109(19):7571–7576, 2012. .
17. E. M. Kramer, N.L. Frazer, and T.I. Baskin. Measurement of diffusion within the cell wall in living roots of Arabidopsis thaliana. *J. Exp. Bot.*, 58(11):3005–3015, 2007. .
18. A. Paterlini, I. Belevich, E. Jokitalo, and Y. Helariutta. Computational tools for serial block electron microscopy reveal plasmodesmata distributions and wall environments. *Plant Physiol.*, 184:53–64, 2020. .
19. H. L. Rutschow, T. I. Baskin, and E. M. Kramer. Regulation of solute flux through plasmodesmata in the root meristem. *Plant Physiol.*, 155(4):1817–1826, 2011. .
20. E.E. Deinum, B.M. Mulder, and Y. Benitez-Alfonso. From plasmodesma geometry to effective symplasmic permeability through biophysical modelling. *eLife*, 8:e49000, 2019. .
21. T. Zhu, W. J. Lucas, and T.L. Rost. Directional cell-to-cell communication in the *Arabidopsis* root apical meristem I. An ultrastructural and functional analysis. *Protoplasma*, 203:35–47, 1998. .
22. I. Tal et al. The *Arabidopsis* NPF3 protein is a GA transporter. *Nat. Commun.*, 7:11486, 2016. .
23. N. Wulff et al. An optimized screen reduces the number of GA transporters and provides insights into

nitrate transporter 1/peptide transporter family substrate determinants. *Front. Plant Sci.*, 10, 2019. .
24. J. Binenbaum et al. Gibberellin and abscisic acid transporters facilitate endodermal suberin formation in *Arabidopsis*. *Nat. Plants*, 9:785–802, 2023. .
25. N. E. J. Appleford et al. Function and transcript analysis of gibberellin-biosynthetic enzymes in wheat. *Planta*, 223:568–582, 2006. .
26. J. Williams, A. Phillips, P. Gaskin, and P. Hedden. Function and substrate specificity of the gibberellin 3β-hydroxylase encoded by the *Arabidopsis* GA4 gene. *Plant Physiol.*, 117:559–63, 07 1998.
27. S. Li, M. Yamada, X. Han, U. Ohler, and P. N. Benfey. High-resolution expression map of the *Arabidopsis* root reveals alternative splicing and lincRNA regulation. *Dev. Cell*, 39(4):508–522, 2016. .
28. B. Jacobs, H. Tobi, and G.M. Hengeveld. Linking error measures to model questions. *Ecological Modelling*, 487:110562, 2024. .
29. R. Sager and J.Y. Lee. Plasmodesmata in integrated cell signalling: insights from development and environmental signals and stresses. *J. Exp. Bot.*, 65:6337–58, 2014. .
30. E.M. Bayer and Y. Benitez-Alfonso. Plasmodesmata: Channels under pressure. *Ann. Rev. Plant Biol.*, 75:291–317, 2024. .
31. T. Nawy et al. Transcriptional profile of the *Arabidopsis* root quiescent center. *Plant Cell*, 17(7): 1908–1925, 2005. .
32. L. R. Band and J. R. King. Multiscale modelling of auxin transport in the plant-root elongation zone. *J. Math. Biol.*, 65:743–785, 2012. .
33. H. Sze. H^+-translocating ATPases: Advances using membrane vesicles. *Annu. Rev. Plant Physiol.*, 36 (1):175–208, 1985. .
34. L. R. Band et al. Systems analysis of auxin transport in the *Arabidopsis* root apex . *The Plant Cell*, 26(3):862–875, 2014. .
35. P. Holzheu et al. An integrative view on vacuolar pH homeostasis in *Arabidopsis thaliana*: Combining mathematical modeling and experimentation. *Plant J.*, 106(6):1541–1556, 2021. .
36. E. M. Kramer. How far can a molecule of weak acid travel in the apoplast or xylem? *Plant Physiol.*, 141(4):1233–1236, 2006. .
37. R. Hedrich, T. D. Mueller, D. Becker, and I. Marten. Structure and function of TPC1 vacuole SV channel gains shape. *Mol. Plant*, 11(6):764–775, 2018. .
38. Mitchison G. J. The dynamics of auxin transport. *Proc. R. Soc. Lond. B.*, 209:489–511, 1980. .
